# Structural Evidence for DUF512 as a Radical *S*-Adenosylmethionine Cobalamin-Binding Domain

**DOI:** 10.1021/acsbiomedchemau.4c00067

**Published:** 2024-10-23

**Authors:** Bo Wang, Amy E. Solinski, Matthew I. Radle, Olivia M. Peduzzi, Hayley L. Knox, Jiayuan Cui, Ravi K. Maurya, Neela H. Yennawar, Squire J. Booker

**Affiliations:** † Department of Chemistry, The Pennsylvania State University, University Park, Pennsylvania 16802, United States; ‡ Department of Biochemistry and Molecular Biology, The Pennsylvania State University, University Park, Pennsylvania 16802, United States; § The Huck Institutes of the Life Sciences, The Pennsylvania State University, University Park, Pennsylvania 16802, United States; ∥ Howard Hughes Medical Institute, The Pennsylvania State University, University Park, Pennsylvania 16802, United States

**Keywords:** radical *S*-adenosylmethionine, DUF512, cobalamin, methylase, crystallography

## Abstract

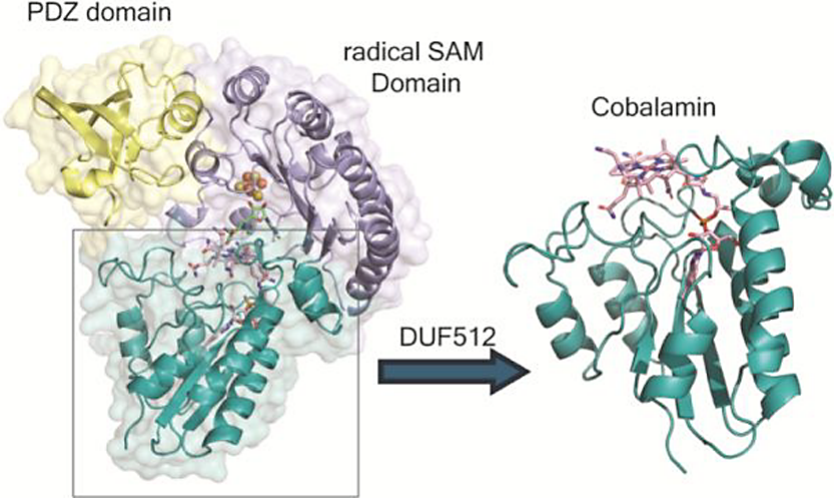

Cobalamin (Cbl)-dependent radical *S*-adenosylmethionine (SAM) enzymes constitute a large subclass of radical SAM (RS) enzymes that use Cbl to catalyze various types of reactions, the most common of which are methylations. Most Cbl-dependent RS enzymes contain an N-terminal Rossmann fold that aids Cbl binding. Recently, it has been demonstrated that the methanogenesis marker protein 10 (Mmp10) requires Cbl to methylate an arginine residue in the α-subunit of methyl coenzyme M reductase. However, Mmp10 contains a Cbl-binding domain in the C-terminal region of its primary structure that does not share significant sequence similarity with canonical RS Cbl-binding domains. Bioinformatic analysis of Mmp10 identified DUF512 (Domain of Unknown Function 512) as a potential Cbl-binding domain in RS enzymes. In this paper, four randomly selected DUF512-containing proteins from various organisms were overexpressed, purified, and shown to bind Cbl. X-ray crystal structures of DUF512-containing proteins from *Clostridium sporogenes* and *Pyrococcus furiosus* were determined, confirming their C-terminal Cbl-binding domains. The structure of the DUF512-containing protein from *C. sporogenes* is the first of an RS enzyme containing a PDZ domain. Its RS domain has an unprecedented β_3_α_4_ core, whereas most RS enzymes adopt a (βα)_6_ core. The DUF512-containing protein from *P. furiosus* has no PDZ domain, but its RS domain also has an uncommon (βα)_5_ core.

## Introduction

Radical *S*-adenosylmethionine (SAM) enzymes constitute one of the largest enzyme superfamilies.^1−6^ These enzymes are often identified by a conserved CX_3_CX_2_C motif, wherein the indicated cysteines coordinate a [Fe_4_S_4_] cluster required for the reductive cleavage of SAM to methionine and a 5′-deoxyadenosyl radical (5′-dA^·^). The 5′-dA^·^, a potent oxidant, is a critical intermediate in all known RS reactions except for TsrM, a Cbl-dependent radical SAM (RS) methylase that methylates an sp^2^-hybridzed carbon center.^4,6−10^ The role of the 5′-dA^·^ is typically to abstract a hydrogen atom from a bound substrate, the first step in more than 100 distinct reaction types identified so far.^1−6^

**Figure 1:**
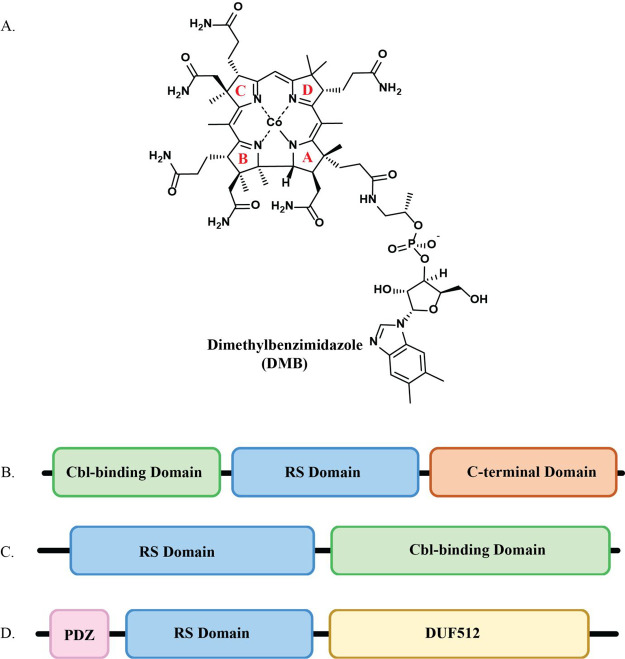
Chemical structure of Cbl and the numbering of the four pyrrole groups in the corrin ring (A). Domain architectures of TsrM/TokK (B), Mmp10 (C), and the DUF512 proteins (D).

One large subfamily of RS enzymes requires a cobalamin (Cbl or B_12_, Figure 1A) cofactor in addition to the requisite iron–sulfur (FeS) cluster for its function.^11−17^ Most of these enzymes are methylases that use methylcobalamin (MeCbl) to methylate sp^2^- or sp^3^-hybridized carbon or phosphinate phosphorus centers. However, Cbl-dependent RS enzymes that are not methylases also exist, although the role of Cbl in these reactions has yet to be elucidated. For example, OxsB, the first structurally characterized Cbl-dependent RS enzyme, catalyzes a ring contraction on a 2′-deoxyribose substrate to form the four-membered oxetane ring of oxetanocin-A, an antiviral natural product.^18^

Three structures of Cbl-dependent RS methylases have been determined: those of *Ks*TsrM, TokK, and Mmp10.^19−21^ TsrM methylates C2 of tryptophan during the biosynthesis of the quinaldic acid moiety of thiostrepton A, an antitumor antibiotic, whereas TokK appends three methyl groups iteratively to install an isopropyl group on a carbapenam substrate during the biosynthesis of the carbapenem antibiotic asparenomycin A.^7−10,19,20,22−24^ Lastly, Mmp10 methylates C5 of Arg^285^ of the alpha subunit of methyl coenzyme M reductase (MCR).^25,26^ TokK and Mmp10 methylate sp^3^-hybridized carbons, while TsrM methylates an sp^2^-hybridized carbon using a completely different strategy. Despite differences in their reaction mechanisms, both TsrM and TokK share highly similar domain architectures (Figure 1B), including an N-terminal Rossmann-like Cbl-binding domain, an RS domain with a shortened triose phosphate isomerase (TIM) barrel harboring the three cysteine residues that ligate the FeS cluster, and a C-terminal domain for substrate and Cbl binding.^19,20,27^

A sequence alignment of Mmp10, TsrM, TokK, and other known Cbl-dependent RS methylases showed that Mmp10 does not contain an N-terminal Cbl-binding domain, which impeded its identification as a Cbl-binding RS enzyme. However, Radle et al. demonstrated that Mmp10 indeed requires Cbl for catalysis, suggesting that the Cbl-binding domain might be in the C-terminal region of the enzyme, which was subsequently confirmed by X-ray crystallography (Figure 1C).^21,26^ While analyzing the sequence of Mmp10 across several bioinformatics databases, Radle et al. observed one annotation from the Integrated Microbial Genomes and Microbiomes (IMG/M) database that suggested the protein contains a DUF512 domain. However, Mmp10 is not grouped into the DUF512-containing family by InterPro or Pfam databases.^26,28^ Like in Mmp10, DUF512 domains are almost always located C-terminal to an RS domain, suggesting DUF512 is a Cbl-binding domain (Figure 1D). In the study herein, four DUF512-containing proteins from various organisms were randomly selected, overexpressed, and reconstituted with Cbl. Each protein catalyzes the transfer of a methyl group to bound Cbl to generate MeCbl. X-ray crystal structures of DUF512-containing proteins from *Clostridium sporogenes* and *Pyrococcus furiosus* show unique architectural features and confirm the presence of a C-terminal Cbl-binding domain like that of Mmp10. There are ∼6,000 sequences in the DUF512 family, and these results suggest they should be grouped into the Cbl-dependent RS methylase subfamily.

## Materials and Methods

### Materials

All commercial materials were used as received unless otherwise noted. *N*-(2-Hydroxyethyl)-piperazine-*N*′-(2-ethanesulfonic acid) (HEPES), 2-amino-2-hydroxymethyl-1,3-propanediol (also known as tris(hydroxymethyl)aminomethane (Tris base)) were purchased from Fisher Scientific. Imidazole was purchased from J. T. Baker Chemical Co. Potassium chloride and glycerol were purchased from EMD Chemicals. 2-Mercaptoethanol, *S*-adenosylhomocysteine (SAH), and 5′-deoxyadenosine (5′-dA) were purchased from MilliporeSigma. Kanamycin, ampicillin, isopropyl β-d-1-thiogalactopyranoside (IPTG), and dithiothreitol (DTT) were purchased from Gold Biotechnology. Ni-NTA resin was purchased from Qiagen. Plasmid DNA isolation kits were purchased from Macherey-Nagel (Dueren, Germany). SAM was synthesized and purified as described previously.^29^ All other chemicals and materials were of the highest grade available and were from MilliporeSigma. Perfluoropolyether cryo oil and crystal screening conditions were purchased from Hampton Research. Malonic acid, imidazole, boric acid (MIB) buffer was purchased from Jena Bioscience.

### General Methods

UV–visible spectra were recorded on a Cary 50 spectrometer from Varian (Agilent Technologies, Santa Clara, CA) using the WinUV software package to control the instrument. High-resolution mass spectrometry (HRMS) was conducted on a Thermo Scientific Q Exactive HF-X hybrid quadrupole-Orbitrap mass spectrometer in line with a Vanquish UPLC. Data were collected and processed using Thermo Scientific Xcalibur 4.2.47 software.

### Plasmids and Strains

The genes for three bacterial DUF512 proteins from *Acinetobacter sp. CAG: 196_36_41* (UniProt ID: A0A1Q6TSJ3, *ac*DUF512), *Clotridioides difficile* ATCC 9689 = DSM 1296 (UniProt ID: A0A125V8J6, *cd*DUF512), *Clostridium sporogenes* (UniProt ID: A0A1J1D2P3, *cs*DUF512), and one archaeal DUF512 protein from *Pyrococcus furiosus* (strain ATCC 43587) (UniProt ID: Q8U2I5, *pf*DUF512) were codon-optimized for expression in *E. coli* (sequences in SI) and synthesized by GeneArt (ThermoFisher, Carlsbad, CA). Each gene was subcloned into the pSUMO vector using *Nde*I and *Xho*I restriction sites. The resulting plasmids were used to transform *E. coli* BL21 (DE3) competent cells containing plasmid pDB1282.

### Overexpression of DUF512 Proteins

A 200 mL starter culture in Luria–Bertani (LB) broth containing 50 mg/L kanamycin and 100 mg/L ampicillin was inoculated with a single colony and shaken at 37 °C and 250 rpm for 12 h. Twenty-five mL of the starter culture was used to inoculate 4 L of M9 medium containing 50 mg/L kanamycin and 100 mg/L ampicillin and incubated at 37 °C with shaking at 180 rpm. Expression of the genes encoded on plasmid pDB1282 was induced at an OD_600_ of 0.3 with arabinose (8.0 g, 0.2% w/v). At an OD_600_ of 0.6, the flasks were placed in an ice–water bath for 0.5 h. Once cooled, IPTG was added to a final concentration of 0.5 mM, and iron chloride was added to a final concentration of 25 μM. The cultures were incubated overnight for 18 h at 18 °C with shaking at 180 rpm. The cells were harvested, flash-frozen in liquid nitrogen, and stored at −80 °C until use.

### Purification of DUF512 Proteins

For purification, 30 g of frozen cell paste was resuspended in 150 mL of lysis buffer (50 mM HEPES, pH 7.5, 300 mM KCl, 10% (v/v) glycerol, and 10 mM BME) containing lysozyme (150 mg) and DNaseI (15 mg). Resuspended cells were incubated on ice and subjected to six 45-s sonic bursts (35% output) on a QSonica instrument in a Coy anaerobic chamber with 8 min intermittent pauses. The lysate was centrifuged for 1 h at 50,000 *g* at 4 °C. The resulting supernatant was loaded onto Ni-NTA resin equilibrated in the lysis buffer. The resin was washed with 200 mL of the lysis buffer before eluting the SUMO–DUF512-containing proteins with elution buffer (50 mM HEPES, pH 7.5, 300 mM KCl, 250 mM imidazole, 10% (v/v) glycerol, and 10 mM BME). The pooled eluate was concentrated by ultracentrifugation using an Amicon Ultra centrifugal filter unit with a 10 kDa molecular weight cutoff membrane. The buffer of the eluate was exchanged into cleavage buffer (75 mM HEPES, pH 7.5, 300 mM KCl, 10 mM imidazole, 15% (v/v) glycerol, and 1.5 mM BME) using a PD-10 column (GE Healthcare). Ulp1 (0.5 mL, 8 mg/mL, 4 mg), cobalamin (3.0 mg), FeCl_3_ (50 μL, 85 mM solution in 50 mM HEPES pH 7.5), and Na_2_S (50 μL, 100 mM solution in water) were added to the buffer-exchanged SUMO–DUF512-protein, and the resulting solution was incubated on ice overnight. The cleavage reaction mixture was loaded on a Ni-NTA column equilibrated with cleavage buffer, and the flow-through was collected, pooled, and concentrated using an Amicon Ultra centrifugal filter unit with a 10 kDa molecular weight cutoff membrane. The resulting DUF512 proteins were further purified by size-exclusion chromatography on a HiPrep 26/60 S200 column housed in an anaerobic chamber, eluting isocratically in S200 buffer (50 mM HEPES, pH 7.5, 400 mM KCl, 10% glycerol, and 4 mM DTT). Fractions containing monomeric DUF512 proteins were pooled, concentrated, frozen in liquid nitrogen, and stored in liquid nitrogen.

### Crystallization and Structure Determination of *cs*DUF512

All manipulations were carried out in a Coy (Grass Lake, MI) anaerobic chamber at room temperature.

The protein solutions contained 12 mg/mL *cs*DUF512, 20 mM HEPES, pH 7.5, and 4 mM 5′-[*N*-[3*S*]-3-amino-carboxypropyl]-*N*-methylamino]-5′-deoxyadenosine (AzaSAM). All crystals were large brown plates obtained within a week using the hanging-drop vapor-diffusion method. *cs*DUF512 crystals formed in 100 mM MIB buffer, pH 5.0 (sodium malonate, imidazole, and boric acid in the molar ratios 2:3:3), 25% (w/v) PEG1500 as the precipitating reagent by mixing 2 μL of the protein solution with 2 μL of the precipitating reagent in the hanging drop and equilibrating against 0.6 mL of aqueous lithium chloride solution (0.5 M). Crystals were briefly dipped in cryoprotectant (perfluoropolyether cryo oil), mounted on nylon loops, and flash-cooled in liquid nitrogen.

X-ray diffraction data sets were collected at the General Medical Sciences and Cancer Institutes Collaborative Access Team (GM/CA-CAT) at the Advanced Photon Source, Argonne National Laboratory. All data sets were processed using the HKL2000 package, and structures were determined by molecular replacement using the program PHASER.^30−33^ Model building and refinement were performed with Coot and phenix.refine.^30,34^ Data collection and refinement statistics are shown in Table S1. Figures were prepared using PyMOL.^35,36^

Diffraction data were collected at λ = 1.0332 Å with 360° of data measured using a 0.5° oscillation range to 1.68 Å resolution (Table S1). The data was processed to 1.68 Å, limited by the detector distance. Alpha fold model (https://alphafold.ebi.ac.uk/entry/A0A1J1D2P3) was used for molecular replacement using Phenix Phase-MR.^30^ Iterative manual model building and refinement were performed in Coot and Phenix.^30,34^*R*_free_ flags were determined by Phenix using the default settings (10% or up to 2000 reflections).

Geometric restraints for AzaSAM and cobalamin were obtained from the Grade Web Server (Global Phasing). In the later stages of refinement, Translation/Libration/Screw (TLS) parameters were additionally used. The final AzaSAM-bound structure consists of residues 1–443 (of 444 residues), one [4Fe-4S] cluster, one cobalamin, one AzaSAM, two imidazole molecules, two boric acid molecules, and one glycerol molecule in the model. The final structure also contains 497 water molecules. Analysis of the Ramachandran statistics showed that 98% of residues are in favored regions, with the remaining 2% in allowed region. Data collection and refinement statistics are shown in Table S1.

Restraints for [4Fe-4S] clusters were based on the structure of *M. thermoacetica* carbon monoxide dehydrogenase/acetyl-CoA synthase (PDB ID: 3I01).^37^

### Crystallization and Structure Determination of *pf*DUF512

All manipulations were carried out in a Coy (Grass Lake, MI) anaerobic chamber at room temperature.

The protein solutions contained 20 mg/mL *pf*DUF512, 20 mM HEPES, pH 7.5, and 5 mM SAH. All crystals were brown cubes obtained within 72 h using the hanging-drop vapor-diffusion method. *pf*DUF512 crystals formed in 100 mM MMT buffer (DL-malic acid, MES and Tris base in the molar ratio 1:2:2), pH 6.0, 30% (w/v) PEG1500 as the precipitating reagent by mixing 2 μL of the protein solution with 2 μL of the precipitating reagent in the hanging drop and equilibrating against 0.5 mL of a precipitating solution. Crystals were briefly dipped in cryoprotectant (perfluoropolyether cryo oil), mounted on nylon loops, and flash-cooled in liquid nitrogen.

X-ray diffraction data sets were collected at the Advanced Light Source (ALS) of the Berkeley Center for Structural Biology. All data sets were processed using the HKL2000 package, and structures were determined by molecular replacement using the program PHASER.^30−33^ Model building and refinement were performed with Coot and phenix.refine.^30,34^ Data collection and refinement statistics are shown in Table S1. Figures were prepared using PyMOL.^35,36^

Diffraction data for were collected at λ = 1.0377 Å with 360° of data measured using a 0.25° oscillation range to 1.95 Å resolution (Table S1). Alpha fold model (https://alphafold.ebi.ac.uk/entry/Q8U2I5) was used for molecular replacement using Phenix Phase-MR.^30^ Iterative manual model building and refinement were performed in Coot and Phenix.^30,34^*R*_*free*_ flags were determined by Phenix using the default settings (10% or up to 2000 reflections). Geometric restraints for SAH and 5′dA were obtained from the Grade Web Server (Global Phasing). In the later stages of refinement, Translation/Libration/Screw (TLS) parameters were additionally used. The final SAH-bound structure consists of residues 1–357 (of 358 residues) with an additional histidine residue at the N-terminal from the SUMO-tag, one [4Fe-4S] cluster, one cobalamin, one SAH molecule, one 5′dA molecule, and one potassium ion in the model. The final structure also contains 303 water molecules. Analysis of the Ramachandran statistics showed that 97% of residues are in favored regions, with the remaining 3% in allowed regions. Data collection and refinement statistics are shown in Table S1.

Restraints for [4Fe-4S] clusters were based on the structure of *M. thermoacetica* carbon monoxide dehydrogenase/acetyl-CoA synthase (PDB ID: 3I01).^37^

### Sequence Similarity Network (SSN) Generation

The SSN was generated to include both known Cbl-dependent RS enzymes and the newly identified DUF512 subgroup on Enzymefunction.org. A list of accession codes was compiled, starting with Megacluster 2–1 which was downloaded from RadicalSAM.org (https://radicalsam.org/explore.php?id=cluster-2-1&v=3.0). Additional sequences were included for the following functionally characterized enzymes (with Uniprot accession codes): SlTsrM (C0JRZ9), KsTsrM (E4N8S5), GenK (Q70KE5), GenD1 (Q2MG55), PhpK (A0A0M3N271), Fom3 (Q56184), CysS (A0A0H4NV78), OxsB (O24770), PoyC (J9ZXD6), swb7 (D2KTX6), ArgMT (Q8THG6), ThnK (F8JND9), TokK (A0A6B9HEI0), BchE (Q7 X 2C7), CouN6 (A0A1H2F7M3) and CloN6 (Q9F8U1). The Enzyme Function Initiative (EFI) enzyme similarity tool (EFI-EST) (https://efi.igb.illinois.edu) was used to perform an all-by-all BLAST analysis with the list of accession codes. We utilized the “Protein Family Addition Options” to add the DUF512 Pfam (PF04459) to the analysis. After initial processing, an alignment score of 70 was chosen for the network which contained 15,692 sequences. The representative node network (50% ID) contained 11,750 nodes and 1,160,343 edges. This network was downloaded and opened using Cytoscape, where known Cbl-dependent radical SAM enzymes and the newly identified DUF512-containing enzymes were labeled.

### Colored SSN Generation and Chemically Guided Functional Profiling (CGFP) Analysis

An SSN was created on EFI that included the DUF512 Pfam (∼6000 sequences). The initial SSN, which contained 3018 representative nodes, was then filtered to an edge percent identify of 50% using Cytoscape. The SSN was submitted to the Genome Neighborhood Tool (GNT) on EFI. This analysis produced a colored (labeled) SSN file which was then resubmitted to EFI for CGFP analysis. The quantification step utilized all available metagenomes on EFI. The resulting heatmap was downloaded directly from EFI.

## Results

### Selected DUF512-Containing Proteins Bind Cobalamin

To assess whether DUF512-containing proteins bind Cbl, three bacterial DUF512-containing proteins from *Acinetobacter sp. CAG: 196_36_41* (UniProt ID: A0A1Q6TSJ3, *ac*DUF512), *Clostridioides difficile* ATCC 9689 = DSM 1296 (UniProt ID: A0A125V8J6, *cd*DUF512), and *Clostridium sporogenes* (UniProt ID: A0AAE4Z2Q4, *cs*DUF512), and one archaeal DUF512-containing protein from *Pyrococcus furiosus* (strain ATCC 43587) (UniProt ID: Q8U2I5, *pf*DUF512) were selected for biochemical and structural characterization. The corresponding genes were optimized for expression in *E. coli*, synthesized by GenScript, and ligated into a SUMO vector to produce an N-terminal SUMO-tagged protein, where the SUMO tag can be removed by treatment with the Ulp1 protease. Each protein was purified to ≥95% homogeneity (Figure S1). Reconstitution of the proteins’ Cbl and FeS cofactors was performed simultaneously with the SUMO cleavage reaction. Iron analysis of the purified proteins showed that the iron numbers increased from 1.84 to 2.71 in *cs*DUF512, 1.91 to 2.46 in *pf*DUF512, 2.73 to 3.22 in *ac*DUF52, and 0.81 to 2.83 in *cd*DUF512.^38^ Each of the purified DUF512-containing proteins exhibits features consistent with the presence of FeS clusters, with a maximum absorbance at 280 nm and a broad feature at 410 nm (Figure S2A). Treatment of the as-isolated (ai) proteins with potassium cyanide does not lead to spectra with peaks at 367 nm, characteristic of cyanocobalamin (CNCbl) (Figure S2B), indicating no Cbl is bound.^39^ The ai proteins were reconstituted with hydroxocobalamin (OHCbl) and repurified by size-exclusion chromatography (S200). The resulting reconstituted proteins exhibit new peaks at ∼320 nm, suggesting the presence of OHCbl (Figure S2C).^8^ Treatment of the reconstituted proteins with potassium cyanide results in sharp peaks at 367 nm, indicating the presence of CNCbl (Figure S2D). Quantification of CNCbl (ε = 30,800 M^–1^/cm at 367 nm) shows 0.75 (*ac*DUF512), 0.82 (*cs*DUF512), 0.96 (*cd*DUF512), and 0.80 (*pf*DUF512) equivalents per protein.^39^ These results demonstrate that all selected DUF512-containing proteins are Cbl-binding proteins.

Cbl-dependent RS methylases use SAM to methylate cob(I)alamin to generate MeCbl during catalysis, forming SAH as a coproduct. Upon incubating the four DUF512 proteins with SAM and titanium(III) citrate (reductant) for 5 min, OHCbl is converted into MeCbl (Figure S3A). The quantification of SAH generated in the *cs*/*ac*/*cd*DUF512 reactions is consistent with the complete conversion of cob(I)alamin to MeCbl (Figure S3B). To assess whether these proteins catalyze the abortive cleavage of SAM to methionine and a 5′-dA^·^, 5′-deoxyadenosine (5′-dA) was also quantified (Figure S3C). All four DUF512 proteins give a small but detectable amount of 5′-dA. These observations indicate that these DUF512 proteins are RS methylases. Previous studies of Cbl-dependent RS methylases were hampered by poor solubility during overproduction, presumably due to the lack of intracellular Cbl as a cofactor to support their correct folding.^40^ Lanz et al. reported that coexpressing Cbl-binding RS proteins with genes in the *E. colibtu* operon, encoding proteins involved in Cbl uptake and trafficking, substantially increased the level of soluble purified Cbl-dependent RS methylases.^41^ However, unlike most previously studied Cbl-dependent RS methylases, the selected DUF512-containing proteins could be isolated in a soluble form without coexpression with the *btu* operon and supplementing with Cbl. This observation was also true for Mmp10.^21,26^ When *cs*DUF512 is coexpressed with the *btu*-associated genes, a similar yield and purity of the protein (Figure S4A) is obtained. However, analysis of the protein shows multiple forms of bound Cbl: 25% OHCbl, 37% MeCbl, and 38% adenosylcobalamin (AdoCbl) (Figure S4B). By contrast, without coexpressing with the *btu*-associated genes, DUF512-containing proteins do not contain significant amounts of bound Cbl upon isolation, which allows them to be reconstituted with a single form of Cbl to obtain homogeneous proteins.

### *cs*DUF512 Is the First Structurally Characterized Radical SAM Enzyme with a PDZ Domain

**Figure 2:**
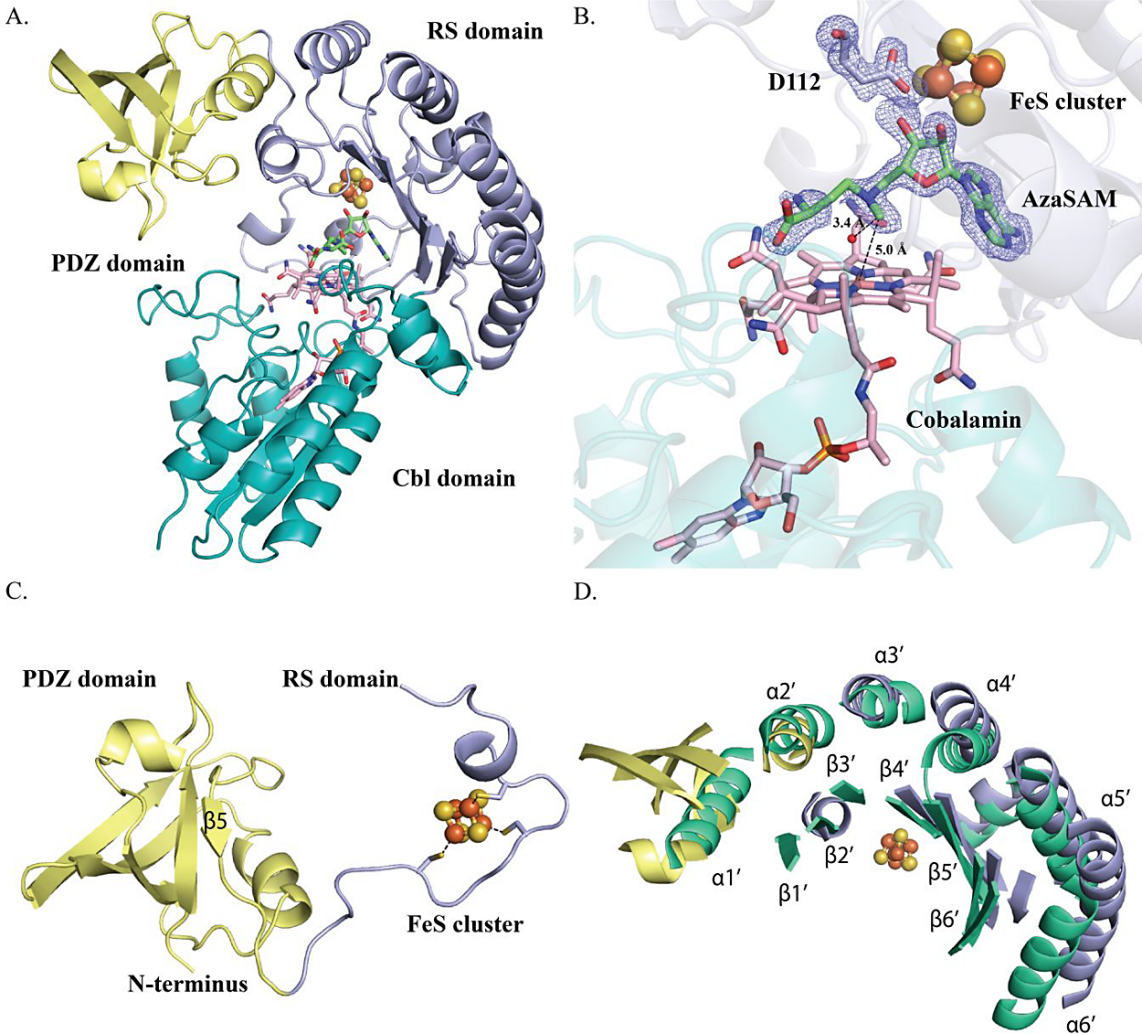
(A) Domain architecture of *cs*DUF512 with AzaSAM, FeS cluster, and Cbl. PDZ domain: yellow, RS domain: light blue, Cbl-binding domain: teal. (B) Closeup of AzaSAM, FeS cluster, Cbl, and D112 within the *cs*DUF512 structure. The side chain of D112 coordinates the unique iron of the FeS cluster. Fo-Fc omit electron density maps (blue mesh) of D112 and AzaSAM, contoured at 4.0 σ and 2.5 σ, respectively, are depicted. For clarity, the major conformer of AzaSAM is shown. (C) PDZ domain and cluster loop of *cs*DUF512. The cluster loop is preceded by β_5_ of the PDZ domain. (D) Superimposition of the RS domain of Mmp10 (green) onto the PDZ (yellow) and RS (light blue) domains of *cs*DUF512. For clarity, loops in both structures were removed. The labeling of helices and strands is based on the (βα)_6_ TIM barrel of Mmp10. The RS domain of *cs*DUF512 lacks the corresponding first three β-strands (β1′, β2′, and β3′) and two α-helices (α1′ and α2′) in the RS domain of Mmp10. Mmp10’s α2′ aligns with the long helix of the PDZ domain of *cs*DUF512. The remaining α-helices (α3′−α6′) of the RS domain of Mmp10 align with the four α-helices in the RS domain of *cs*DUF512. The first three β-strands (β1′−β3′) align with a small helix in the *cs*DUF512 structure. The remaining β-strands (β4′−β7′) of the RS domain of Mmp10 align with the first four β-strands of the RS domain of *cs*DUF512.

*cs*DUF512 was crystallized under anoxic conditions in the presence of AzaSAM, and its structure was determined to a resolution of 1.68 Å (Table S1). AzaSAM is a SAM analog containing a nitrogen atom in place of SAM’s sulfur atom, and it has been used as a SAM surrogate in several structural studies.^19,42,43^*cs*DUF512 is composed of three domains (Figure 2A,B, topology diagram in Figure S5A), the first of which (residues 1–80) is a PDZ domain.^44^ Proteins with PDZ domains are found predominantly in metazoans; the PDZ abbreviation is derived from three metazoan proteins (PSD-95, DLG, and ZO-1).^44^ Although less prevalent, PDZ domains are also present in bacteria, where studies have predominantly focused on the high-temperature requirement A (HTRA) family, such as DegP, DegS, and DegQ.^45^ The PDZ domain has a compact globular fold comprising five antiparallel β-strands and two α-helices.^46^ A canonical arrangement of a PDZ domain is β1-β2-β3-α1-β4-α2-β5, which is found in the majority of human PDZ domain structures (Figure S6A).^47^ The arrangement (β3-α1-β4-α2-β5-β1-β2) of *cs*DUF512’s PDZ domain (Figure S6B) is different from this arrangement but identical to that of the GRASP55 PDZ domain (PDB ID: 4EDJ), involved in mammalian Golgi biogenesis.^48^ A striking difference we observed is the location of PDZ domains. As shown in Figure S6C, the bacterial HTRA family, including DegP/Q/S, has its PDZ domain near the C-terminus. However, *cs*DUF512, a bacterial PDZ domain-containing protein, has its PDZ domain near the N-terminus, similar to eukaryotic PDZ domain-containing structures.^46^

PDZ domains usually serve as protein–protein interaction modules that regulate various cellular functions.^46,49^ They were initially recognized as binding the extreme C-terminal residue of their binding partner. The residue inserts into the hydrophobic cleft formed between β2 and α2, where the carboxylate of the residue interacts with a loop between β1 and β2 via a network of hydrogen bonds (Figure S6A).^50^ This loop is also named the carboxylate binding loop, which contains a general sequence of Ψ-Gly-Ψ, wherein Ψ refers to hydrophobic amino acids. We observed the hydrophobic cleft as well as the carboxylate binding loop (Leu72-Gly73-Ile74) in *cs*DUF512’s PDZ domain, indicating that *cs*DUF512 may interact with unknown proteins through its C-terminus. The PDZ domains in DUF512-containing enzymes could function in substrate recognition, but further studies are needed to uncover their true role.

### *cs*DUF512 Has an Incomplete TIM Barrel in Its RS Domain

The second domain (residues 81–266) of *cs*DUF512 has an unusually short and incomplete β_3_α_4_ TIM barrel core (Figure 2A). Most RS enzymes adopt a three-quarter (βα)_6_ TIM barrel as the core of their RS domains.^6,11,12,51^ Pyruvate formate-lyase activating enzyme (PFL-AE), one of the smallest RS enzymes (246 residues in length), with an RS domain only, still adopts a canonical (βα)_6_ fold.^52^ In nearly all structures of RS enzymes, the βα-repeating core of the TIM barrel begins from a strand (β1) followed by a loop (termed the cluster-loop) containing the CX_3_CX_2_C motif that coordinates the FeS cluster.^4^ Interestingly, *cs*DUF512 lacks the starting strand (β1) in its RS domain. Instead, a short strand (β5) from the PDZ domain precedes the cluster-loop (Figure 2C). Subsequently, the first helix (α1) initiates the TIM barrel β_3_α_4_ core. When overlaying the RS domains of Mmp10 and *cs*DUF512 (Figure 2D), the first two α-helices (α1′ and α2′) and the first three β-strands (β1′−β3′) from the N-terminal portion of the RS domain of Mmp10 are missing in the RS domain of *cs*DUF512. Instead, α_2_ of the PDZ domain occupies the space of the second helix of the (βα)_6_ TIM barrel structure, and a short α-helix occupies the space of the first three β-strands of the RS domain of Mmp10. Although *cs*DUF512 has a smaller TIM barrel, sharing some secondary structural features with the adjacent PDZ domain likely complements the incomplete barrel for radical chemistry.

Cys87, Cys91, and Cys94 coordinate the FeS cluster of *cs*DUF512. Notably, an aspartate (D112) residue in the middle of a DDD motif located on the cluster loop is observed to coordinate the unique iron of the FeS cluster (Figure 2B). This DDD motif is highly conserved among all DUF512-containing proteins that harbor PDZ domains (Figure S7A). Superimposing *cs*DUF512 with Mmp10 shows that this DDD_113_ motif partially overlays with the “GGD_90_” motif in the Mmp10 structure, in which D90 interacts with the amino group of SAM, facilitating the binding of SAM to the cluster (Figure S7B).^6,53^ Although AzaSAM is not positioned to bind to the cluster in the *cs*DUF512 structure, D113 of the DDD_113_ motif likely plays a similar role to the Glu in the canonical “GGE” motif. The distance from the carboxylate group of D112 to the unique iron is 2.09 Å, comparable to the 1.7 Å from the OH group of Y115 to the unique iron in Mmp10 and 2.07 Å from the carboxylate group of E273 to the unique iron in TsrM (Figure S8). For Mmp10, both Y115F and Y115A variants exhibit severely impaired SAM cleavage activity and Cbl methylation, consistent with the involvement of Y115 in both S_N_2 and radical chemistry.^21^ The role of E273 coordination in TsrM is less clear. It was proposed to preclude SAM from binding to the cluster for reductive cleavage. However, the E273A variant of TsrM exhibited very low methylation activity and could not generate the 5′-dA^·^ in the presence of reductants. This observation indicates that E273 is crucial for TsrM’s function but is not the critical residue distinguishing between polar and radical chemistries.^24^ Because the substrate for the enzyme is unknown, the role of D112 in *cs*DUF512 is currently unclear.

### Domain Architecture of *pf*DUF512

**Figure 3:**
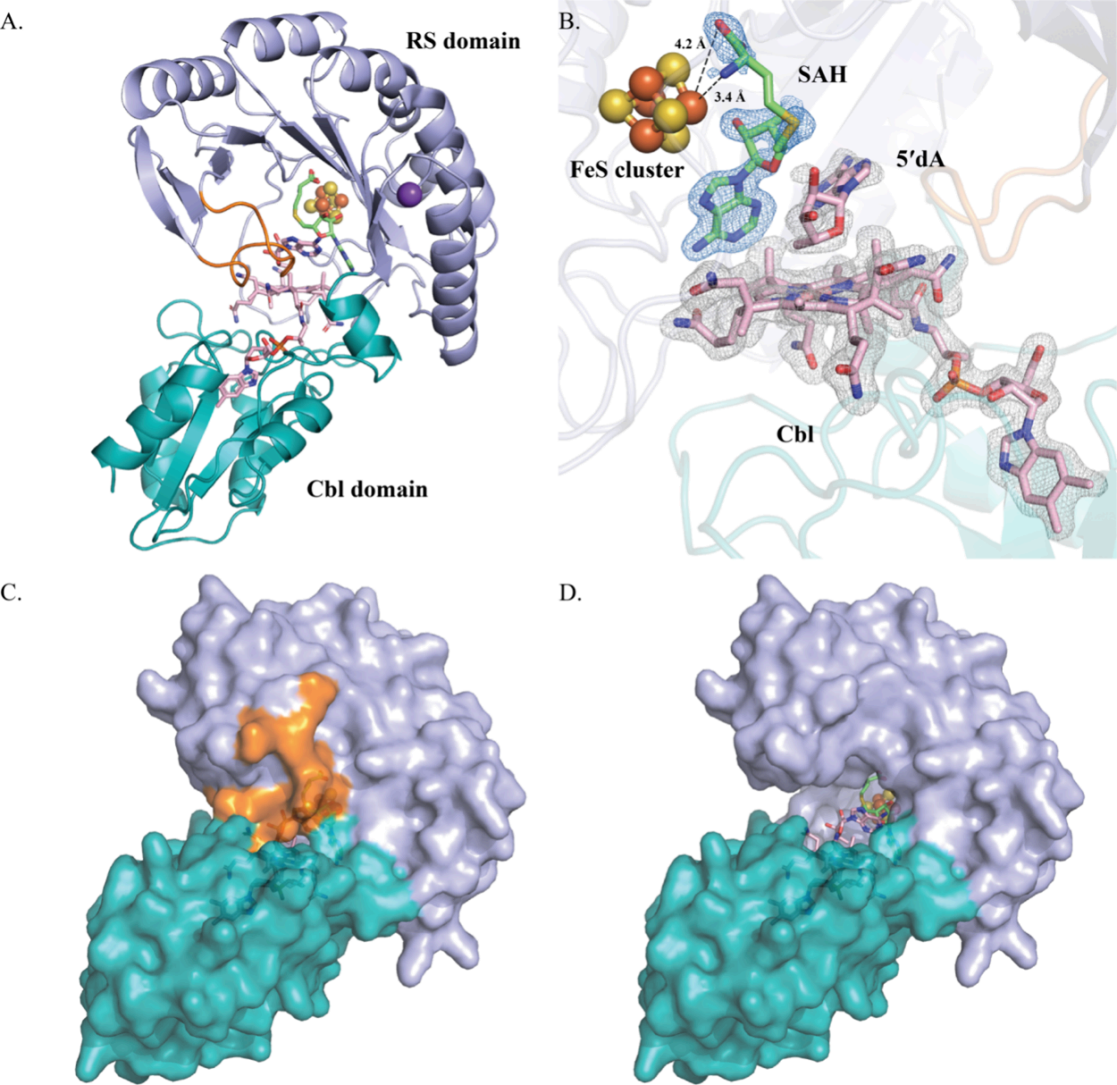
(A) Domain architecture of *pf*DUF512 with SAH, FeS cluster, 5′-dA, and Cbl. L1 within the RS domain is shown in orange. The remainder of the RS domain is shown in light blue. The Cbl-binding domain is shown in teal. The purple sphere represents a potassium ion. (B) Closeup of SAH, FeS cluster, 5′dA, and Cbl within the *pf*DUF512 structure. The 2Fo-Fc maps of 5′-dA and Cbl (gray mesh) and SAH (blue mesh) contoured at 1.0σ are depicted. The distances between the unique iron of the FeS cluster and the amino group or carboxylate group are shown as black dashed lines. (C) *pf*DUF512 is shown as a surface diagram with L1 in orange. (D) Removal of L1 in *pf*DUF512 exposes the active site.

*pf*DUF512 belongs to a subset of DUF512-containing proteins that do not contain PDZ domains. *pf*DUF512 was crystallized under anoxic conditions in the presence of SAH, and its structure was determined to a resolution of 2.07 Å (Table S1). The enzyme contains an N-terminal RS domain (residues 1–222) and a C-terminal Cbl-binding domain (residues 223–357) (Figure 3A,B, topology diagram in Figure S5B). The RS domain has an antiparallel β-sheet (residues 1–14) followed by a loop (L1, residues 15–24) that connects to the TIM barrel core. Although *pf*DUF512’s substrate is unknown, in the current structure, L1 seems to close the active site of *pf*DUF512 by forming stabilizing interactions with the acetamide and DMB tail moieties of Cbl (Figures 3C,D and S9). A similar feature was observed in structures of TsrM: a loop in the C-terminal domain (residues 525–536) moves ∼16.8 Å into the active site, capping the Cbl cofactor and trapping the substrate in the active site.^19^ We suspect that L1 in *pf*DUF512 will likely undergo conformational changes when the substrate binds. The RS domain of *pf*DUF512 has a unique (βα)_5_ TIM barrel core (Figure S10A), with the FeS cluster ligated by Cys39, Cys43, and Cys46. The first three β-strands of *pf*DUF512, compared to the corresponding strands of Mmp10, significantly tilt toward the center of the active site, with movements of 5.5, 5.3, and 6.1 Å, respectively, as calculated from the α-carbons of the corresponding residues (Figure S10B). A molecule of 5′-dA is observed above the cobalt ion of cobalamin. LCMS analysis of *pf*DUF512 used for crystallography shows 44% AdoCbl, 44% OHCbl, and 12% MeCbl bound (Figure S11). The ligand (SAH) in the active site binds in an unusual position, neither ligated to the unique iron of the cluster, as seen in Mmp10, nor close to Cbl, as observed in *cs*DUF512 (Figure 3B). SAH’s ribosyl and methionyl moieties have poor electron density, with very few hydrogen-bonding interactions. By contrast, there is significant electron density for the adenine portion of SAH, which binds approximately in the same position, maintaining a similar hydrogen-bonding network, as observed for Mmp10 and *cs*DUF512. We hypothesize that the presence of 5′-dA above Cbl interferes with the SAH/SAM binding pocket and disrupts the binding of SAH.

### Comparison of Cbl-Binding Domains of DUF512, Mmp10, TsrM, TokK, and OxsB

The Cbl-binding domains of Mmp10 (PDB ID: 7QBS(21); residues 258–410), *cs*DUF512 (residues 267–443), and *pf*DUF512 (residues 223–357) share the same core architecture (Figure S12A–C). The core structure has an extended β-sheet formed by three (*cs*DUF512) or four (*pf*DUF512 and Mmp10) β-strands and α-helices sandwiched between these β-strands in an α/β/α motif. The arrangement mimics the Rossmann fold, which binds Cbl in several Cbl-dependent RS enzymes (Figure S12D–F).^18−20^ The Cbl in *cs*DUF512, *pf*DUF512, and Mmp10 is bound base-off. Like Mmp10, both *cs*DUF512 and *pf*DUF512 have a Leu (L366/*cs*DUF512, L286/*pf*DUF512, L322/Mmp10) in the lower axial position of Cbl, approximately 4.02 and 4.06 Å from the nearest carbon to the cobalt ion, respectively. The Leu residue creates a hydrophobic environment, as in the structures of TokK and Mmp10. TsrM is a Cbl-dependent RS enzyme that catalyzes nonradical methylation on the C2 of the indole ring of L-tryptophan. TsrM has an Arg residue occupying the lower axial position of the Cbl cofactor. It was proposed that the charged guanidium side chain of Arg stabilizes the cob(I)alamin state and destabilizes the methylcob(III)alamin state of the enzyme, which would favor displacement of the strong cob(I)alamin nucleophile by the weakly nucleophilic C2 of Trp.^19^ Therefore, we believe DUF512 proteins likely catalyze radical-dependent methylations. This hypothesis is also supported by the observation of 5′-dA from the abortive cleavage of SAM in the absence of substrates. Figure S13 depicts the environments of Cbl species in *cs*DUF512, *pf*DUF512, Mmp10, OsxB, TsrM, and TokK. The same pattern of Cbl binding is observed in all these Cbl-dependent RS enzymes: the dimethylbenzimidazole moiety of the DMB tails are inserted into a hydrophobic pocket, and N3 of the DMB tails H-bonds to the hydroxyl groups of a threonine residue (Thr323/*cs*DUF512, Thr252/*pf*DUF512, Thr291/Mmp10, and Thr67/TsrM) or a serine residue (Ser72/TokK and Ser84/OxsB).

The most striking difference between the DUF512-containing enzymes (including Mmp10) and the other Cbl-dependent RS enzymes (TsrM, TokK, and OxsB) is the relative location of the Cbl-binding domain and the binding orientations of Cbl. For DUF512-containing enzymes, the FeS cluster is located above rings C and D of Cbl, with a hydrophobic residue between the FeS cluster and Cbl (Y23/Mmp10, F95/*cs*DUF512, and Y47/*pf*DUF512) (Figure S14A–C). For TsrM, TokK, and OxsB, the FeS cluster is located above rings A and B of Cbl. Interestingly, these three enzymes show very different residues between the FeS cluster and Cbl. TokK has a tryptophan (W215) between the FeS cluster and Cbl (Figure S14D), while TsrM has a glutamate (E236) (Figure S14E). By contrast, OxsB has no residue between the cluster and Cbl. These different arrangements may reflect the different reactions they catalyze. TokK and Mmp10, which have the same cluster/hydrophobic residue/Cbl sandwich structure, catalyze radical methylations with a dual use of SAM. Given the same pattern in the structure of *cs*DUF512, it is likely that it also catalyzes a radical methylation. In summary, *cs*/*pf*DUF512 proteins are Cbl-binding enzymes. Despite differences in domain architecture and low sequence identity, the Cbl binding pockets are remarkably similar.

### Hypothetical S_N_2 Methylation of Cobalamin

**Figure 4:**
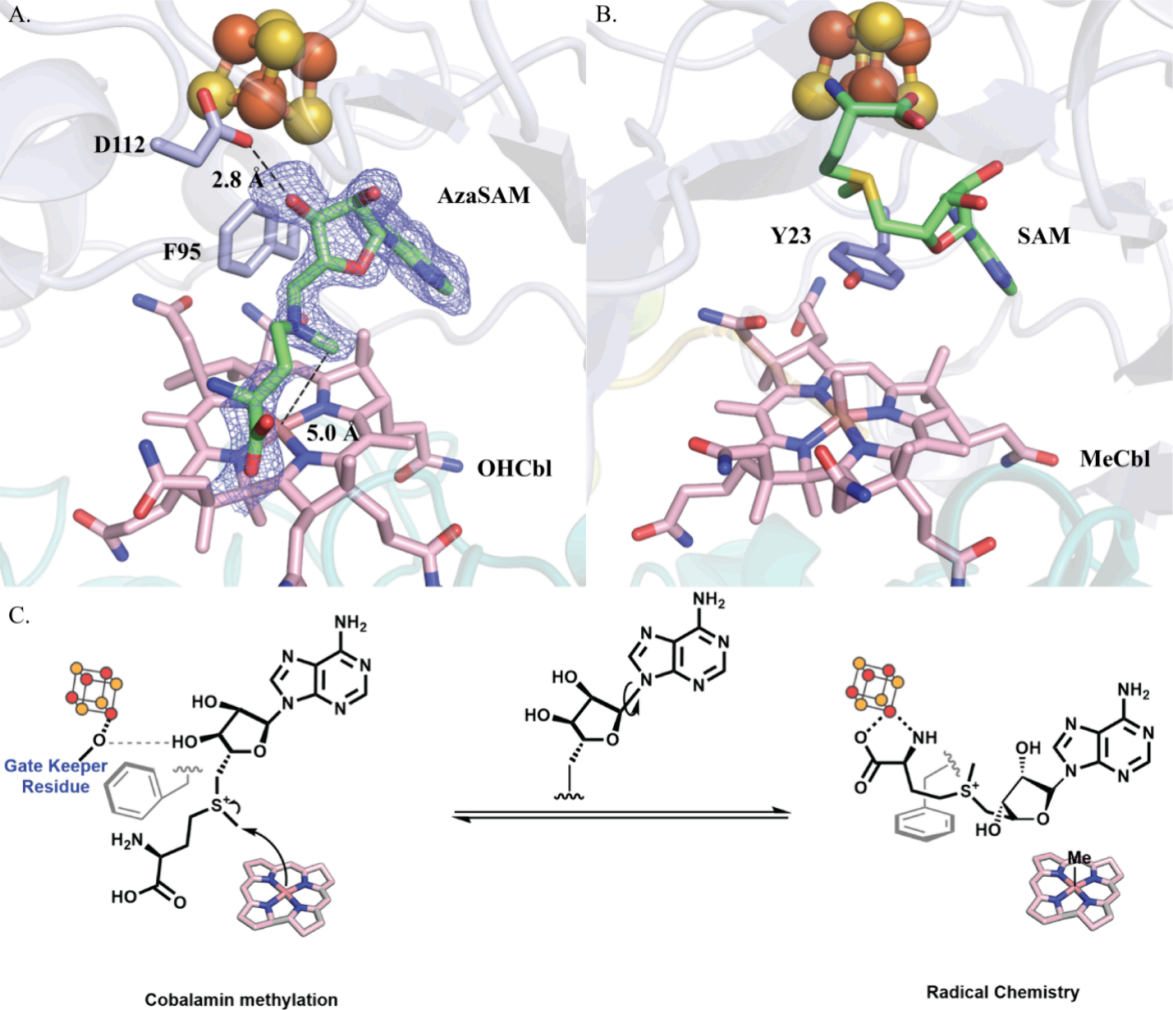
(A) Closeup of D112, F95, AzaSAM, FeS cluster, and Cbl within the *cs*DUF512 structure. Fo-Fc omit electronic density map (blue mesh) of AzaSAM contoured at 2.5 σ is depicted. The phenyl ring of F95 is perpendicular to the corrin ring of Cbl. Such a pose creates space for the methionine moiety of AzaSAM to be flipped down for the polar chemistry of Cbl. 3′–OH of AzaSAM establishes a key hydrogen bond with D112 that coordinates the unique iron of the cluster. (B) Closeup of Y23, SAM, FeS cluster, and MeCbl within the Mmp10 structure (PDB ID 7QBS). The Y23 side chain is parallel to the corrin ring of Cbl and forms π–π stacking with the adenine ring of AzaSAM. (C) Depicted hypothesis of the movements of SAM between radical chemistry and polar methylation of Cbl. Cbl is shown as a pink corrin ring. The hydrophobic residue between the FeS cluster and Cbl is represented by a gray phenyl ring.

The AzaSAM in the *cs*DUF512 structure shows a very different binding pose compared to SAM in the Mmp10 structure (Figure S15).^21^ Some of the canonical motifs for SAM binding, such as a Glu residue interacting with the ribose moiety of SAM and the GXIXGXXE motif, are not observed.^6,53^ Compared to SAM in the Mmp10 structure, the ribose ring of AzaSAM in the *cs*DUF512 structure rotates slightly around the *N*-glycosidic bond. After rotation, the adenosine moiety remains in the same position, but the methyl group of AzaSAM is significantly closer to the cobalt ion (Figure 4A). This distance and orientation suggest an active site arrangement that allows SAM to methylate Cbl to give MeCbl. However, we observe a water molecule above the cobalt ion of Cbl, which may impede the ideal orientation for AzaSAM binding in the *cs*DUF512 structure. Interestingly, the phenyl ring of F95, the hydrophobic residue between the FeS cluster and Cbl in *cs*DUF512, is perpendicular to the corrin ring of Cbl. Corresponding hydrophobic residues in Mmp10 (Y23) are positioned parallel to the corrin ring of Cbl (Figure 4B). The parallel orientation of Y23 in the structure of Mmp10 would clash sterically with SAM if SAM is positioned as observed in the structure of *cs*DUF512 (Figure S16). Thus, we believe this hydrophobic residue between the cluster and Cbl may play a role in distinguishing two orientations of SAM in the active site. Accordingly, we provide a hypothesis for the motions of SAM during catalysis (Figure 4C). For radical methylation, SAM binds to the FeS cluster to be reductively cleaved to a 5′-dA^·^ for substrate H^·^ abstraction. The substrate radical attacks MeCbl, yielding a methylated product and cob(II)alamin, which is converted to cob(I)alamin by an external reductant. To regenerate the MeCbl cofactor, a second SAM molecule binds with its adenine ring in the same position; however, its ribose ring is rotated to position the sulfonium moiety closer to cob(I)alamin for S_N_2 methylation. In this hypothesis, at least two important residues, D112 and F95, are involved in the placement of SAM to distinguish between the two types of chemistry, as suggested by the *cs*DUF512 and Mmp10 structures. Additional mutational and kinetic studies are ongoing to reveal their specific role in these Cbl-dependent RS methylases.

## Conclusions

Herein, we demonstrate that four randomly selected DUF512-containing proteins bind Cbl in their C-terminal domains, thus defining the DUF512 structural motif, present in over 6000 proteins. SAM can methylate the Cbl cofactor in our selected DUF512-containing proteins to generate MeCbl. This observation and the structural features that mimic those of Mmp10 suggest that these proteins are most likely radical-dependent methylases. However, identification of their substrate and subsequent biochemical studies are needed to confirm their biological roles. The X-ray crystal structures of *cs*DUF512 and *pf*DUF512 are highly similar to that of Mmp10. Moreover, a comparison of *cs*DUF512 and *pf*DUF512 with other structurally characterized Cbl-dependent RS enzymes shows they all bind Cbl in a highly similar manner, whether the domains are N-terminal or C-terminal to the RS core domain. However, both structures show unique features not observed in the structures of other RS enzymes, such as a PDZ domain, as found in *cs*DUF512, and the L1 loop that seemingly blocks the active site for *pf*DUF512. Of note, *cs*DUF512 is the first structurally characterized RS enzyme with a PDZ domain. It has an unusually short β_3_α_4_ TIM barrel core in its RS domain, which is completed by linking the PDZ domain to the N-terminus of the RS domain. The AzaSAM in the *cs*DUF512 structure binds in an orientation different from what is observed in Mmp10, which may reflect how SAM methylates Cbl.

**Figure 5:**
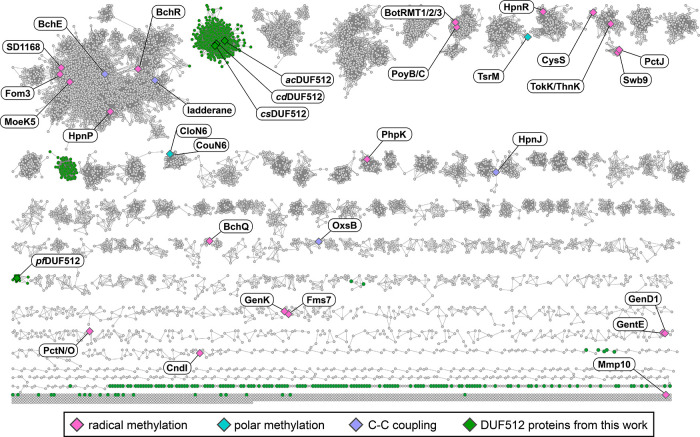
Sequence similarity network (SSN) of Cbl-binding RS enzymes. The SSN was generated to include both known Cbl-dependent RS enzymes and the newly identified DUF512 subgroup with an alignment score of 70. Known and predicted enzyme functions are labeled: radical methylation (pink), C–C coupling (purple), polar methylation (teal), and new DUF512-containing enzymes (green).

The DUF512-containing enzymes are highly prevalent in organisms that reside in the human gut microbiome (Figure S17A,B). There is also a subset of DUF512 proteins present in archaeal organisms. It remains to be seen how the bacterial and archaeal DUF512-containing enzymes are related to each other with respect to their reactions and ecological niches. We have uncovered the “Unknown Function” of the DUF512 protein domain, which is to bind the biological cofactor Cbl. There are ∼6000 sequences in the DUF512 family, and our finding recharacterizes these sequences as Cbl-dependent RS enzymes. With this in mind, we reprocessed a sequence similarity network (SSN) to include all current Cbl-dependent RS enzymes (Figure 5). We observe that the DUF512 nodes (green) do not have any significant sequence similarity with other Cbl-dependent RS enzymes.

## Data Availability

Atomic coordinates and structure factors for the crystal structures reported in this work have been deposited in the Protein Data Bank (PDB) under accession numbers 9CG1 (*cs*DUF512 + AzaSAM) and 9CG2 (*pf*DUF512 + SAH).

## Supporting Information Available

SDS-PAGE of purified DUF512-containing proteins; UV-vis spectra of DUF512-containing proteins; cobalamin methylation of csDUF512 and quantifications of SAH and 5′dA; UV-vis spectra of csDUF512 co-expressed with or without btu gene and cobalamin analysis; topology diagram of csDUF512 and pfDUF512; comparison of PDZ domains; WebLogo sequence alignment of DUF512-containing proteins; distances between unique iron and its interacting residues in the structures of csDUF512, Mmp10, and TsrM; interactions between L1 and cobalamin in the structure of pfDUF512; radical SAM domain of pfDUF512; cobalamin analysis of pfDUF512; comparison of the cobalamin-binding domain in the structures of csDUF512, pfDUF512, Mmp10, TsrM, TokK, and OxsB; hydrogen bond interactions of cobalamin; residues between the FeS cluster and cobalamin in the structure; interaction network of SAM/Mmp10 and azaSAM/csDUF512; overlay of Mmp10 and csDUF512 structures; analysis of DUF512 proteins in the human microbiome; sequences of four DUF512-containing proteins; crystallographic data; and gene sequences of the four DUF512-containing proteins (PDF)

## Abbreviations

5′-dA: 5′-deoxyadenosine
5′-dA^·^: 5′-deoxyadenosyl radical
ai: as-isolated
aa: amino acid
AdoCbl: adenosylcobalamin
AzaSAM: 5′-[*N*-[3*S*]-3-amino-carboxypropyl]-*N*-methylamino]-5′-deoxyadenosine
B_12_: cobalamin
Cbl: cobalamin
CNCbl: cyanocobalamin
FeS: iron–sulfur
HTRA: high-temperature requirement A
LCMS: liquid chromatography with detection by mass spectrometry
MCR: methyl coenzyme M reductase
MeCbl: methylcobalamin
MRM: multiple reaction monitoring
OHCbl: hydroxocobalamin
PDZ: PSD-95, DLG, and ZO-1
RS: radical *S*-adenosylmethionine
SAH: *S*-adenosylhomocysteine
SAM: *S*-adenosylmethionine
