# Supplementary material for: Structural Evidence for DUF512 as a Radical S-Adenosylmethionine Cobalamin-Binding Domain
Source: ACS Bio Med Chem Au. 2024 Oct 23;4(6):319–30. doi: 10.1021/acsbiomedchemau.4c00067 (PMC11659888; doi:10.1021/acsbiomedchemau.4c00067)
Supplement: Supplementary file 1 — bg4c00067_si_001.pdf [file bg4c00067_si_001.pdf]

Supporting Information for:

Structural Evidence for DUF512 as a Radical S-Adenosylmethionine Cobalamin-Binding Domain

Bo Wang[1], Amy E. Solinski[1], Matthew I. Radle[1,#], Olivia M. Peduzzi[1], Hayley L. Knox[1,$], Jiayuan Cui[1], Ravi K. Maurya[1], Neela H. Yennawar[3], and Squire J. Booker[1,2,4,]*

[1] Department of Chemistry, The Pennsylvania State University, University Park, Pennsylvania 16802, USA

[2] Department of Biochemistry and Molecular Biology, The Pennsylvania State University, University Park, Pennsylvania 16802, USA

[3] The Huck Institutes of the Life Sciences, The Pennsylvania State University, University Park, Pennsylvania 16802, USA

[4] Howard Hughes Medical Institute, The Pennsylvania State University, University Park, Pennsylvania 16802, USA

* Email address: sjb14@psu.edu

Present Addresses:

[#] AstraZeneca, 4344 Langston Blvd Arlington, Virginia 22207, USA

[$] Department of Chemistry, Boston University, Boston, Massachusetts 02215, USA.

TABLE of CONTENTS

Page 2-3. Table of contents

Page 4. Figure S1. SDS-PAGE of purified DUF512-containing proteins

Page 5. Figure S2. UV-vis spectra of DUF512-containing proteins

Page 6-7. Figure S3. Cobalamin methylation of *cs*DUF512 and quantifications of SAH and 5′dA

Page 8. Figure S4. UV-vis spectra of *cs*DUF512 co-expressed with or without *btu* gene and cobalamin analysis

Page 9. Figure S5. Topology diagram of *cs*DUF512 and *pf*DUF512

Page 10. Figure S6. Comparison of PDZ domains

Page 11. Figure S7. WebLogo sequence alignment of DUF512-containing proteins with PDZ-domains and comparison of GGD domain (Mmp10) and DDD domain (*cs*DUF516)

Pages 12. Figure S8. The distances between unique iron and its interacting residues in the structures of *cs*DUF512, Mmp10, and TsrM

Pages 13. Figure S9. Interactions between L1 and cobalamin in the structure of *pf*DUF512

Pages 14. Figure S10. Radical SAM domain of *pf*DUF512

Pages 15. Figure S11. The cobalamin analysis of *pf*DUF512

Pages 16-17. Figure S12. Comparison of the cobalamin-binding domain in the structures of *cs*DUF512, *pf*DUF512, Mmp10, TsrM, TokK, and OxsB

Pages 18-19. Figure S13. Hydrogen bond interactions of cobalamin with *cs*DUF512, *pf*DUF512, Mmp10, OxsB, TsrM, and TokK

Pages 20-21. Figure S14. The residues between the FeS cluster and cobalamin in the structure of Mmp10, *cs*DUF512, *pf*DUF512, TokK, and TsrM

Pages 22. Figure S15. Interaction network of SAM/Mmp10 and azaSAM/*cs*DUF512

Pages 23. Figure S16. Overlay of Mmp10 and *cs*DUF512 structures showing important residues (F95/Y23) for the two binding poses of SAM

Pages 24. Figure S17. Analysis of DUF512 proteins in the human microbiome

Pages 25-28. Sequences of four DUF512-containing proteins

Pages 29. Table S1. Crystallographic data

Pages 31. References

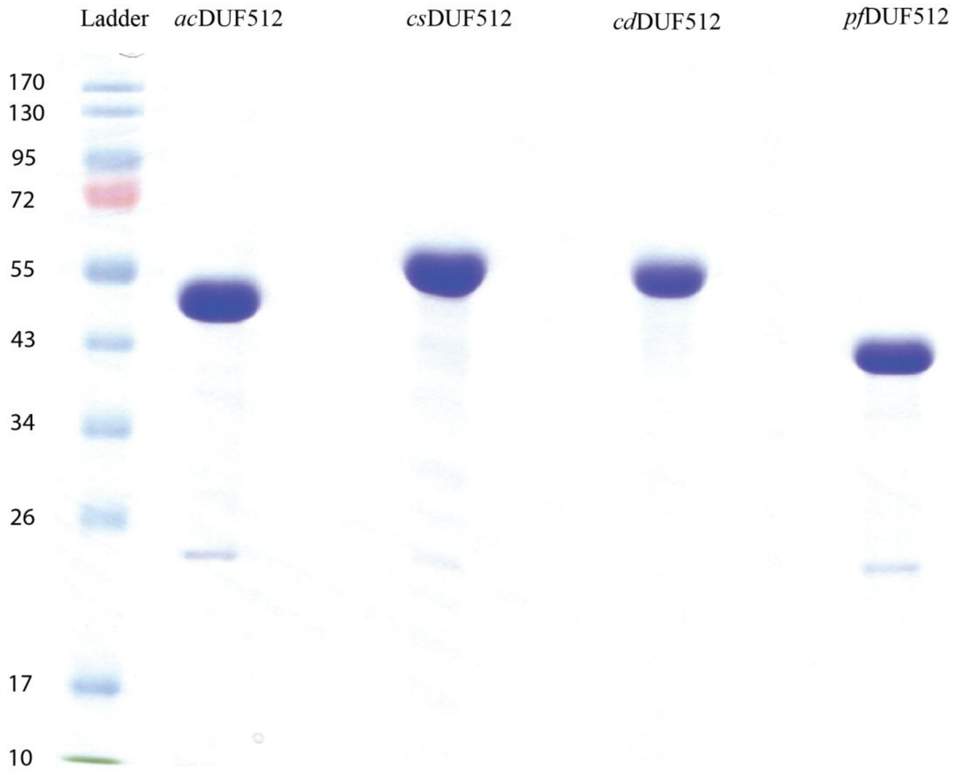

Figure S1. SDS-PAGE of purified DUF512-containing proteins.

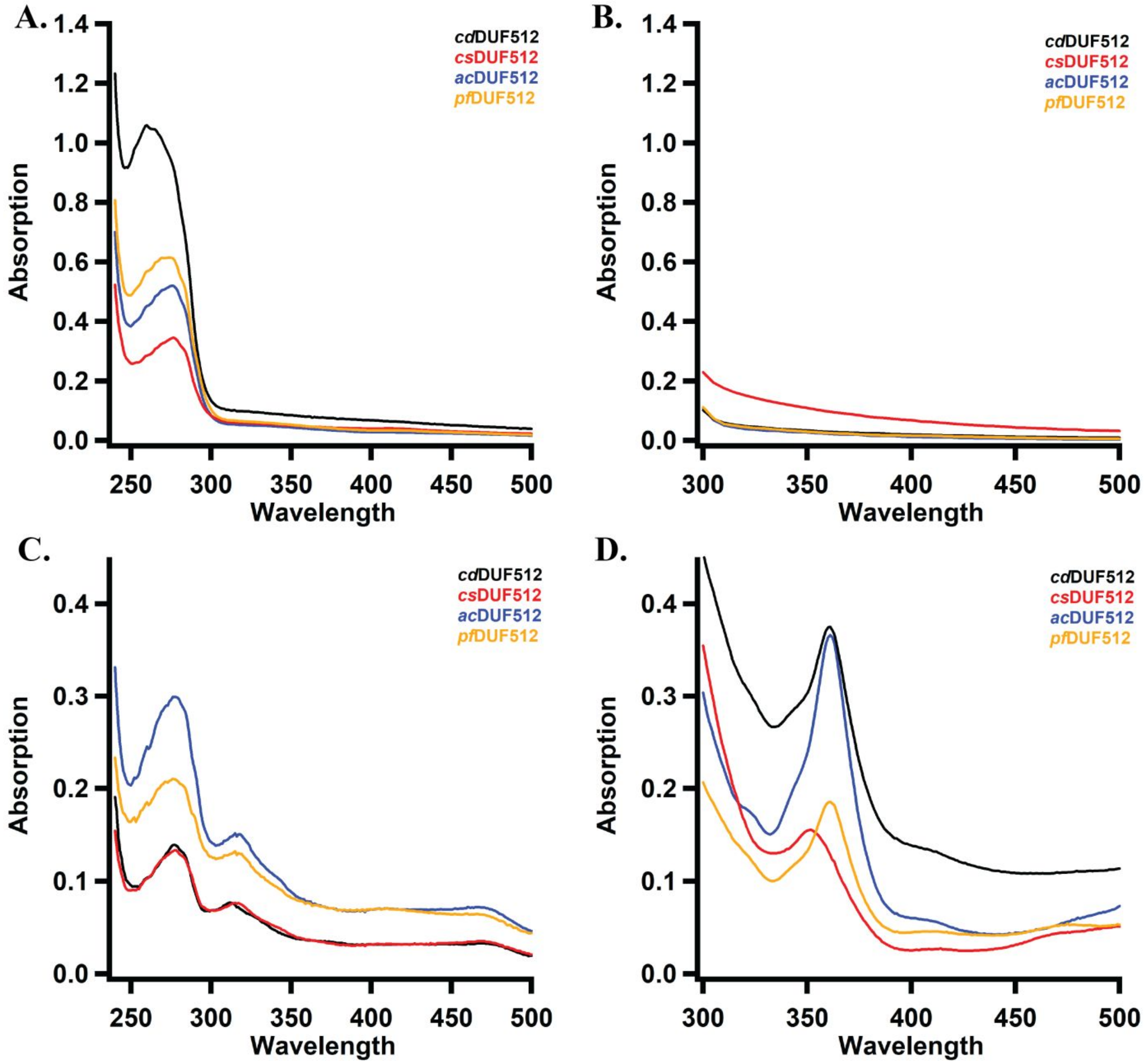

Figure S2. UV-vis spectra of four selected DUF512-containing proteins. **A.** as-isolated; **B.** as-isolated and KCN-treated; **C.** OHCbl reconstituted; **D.** OHCbl reconstituted and KCN-treated.

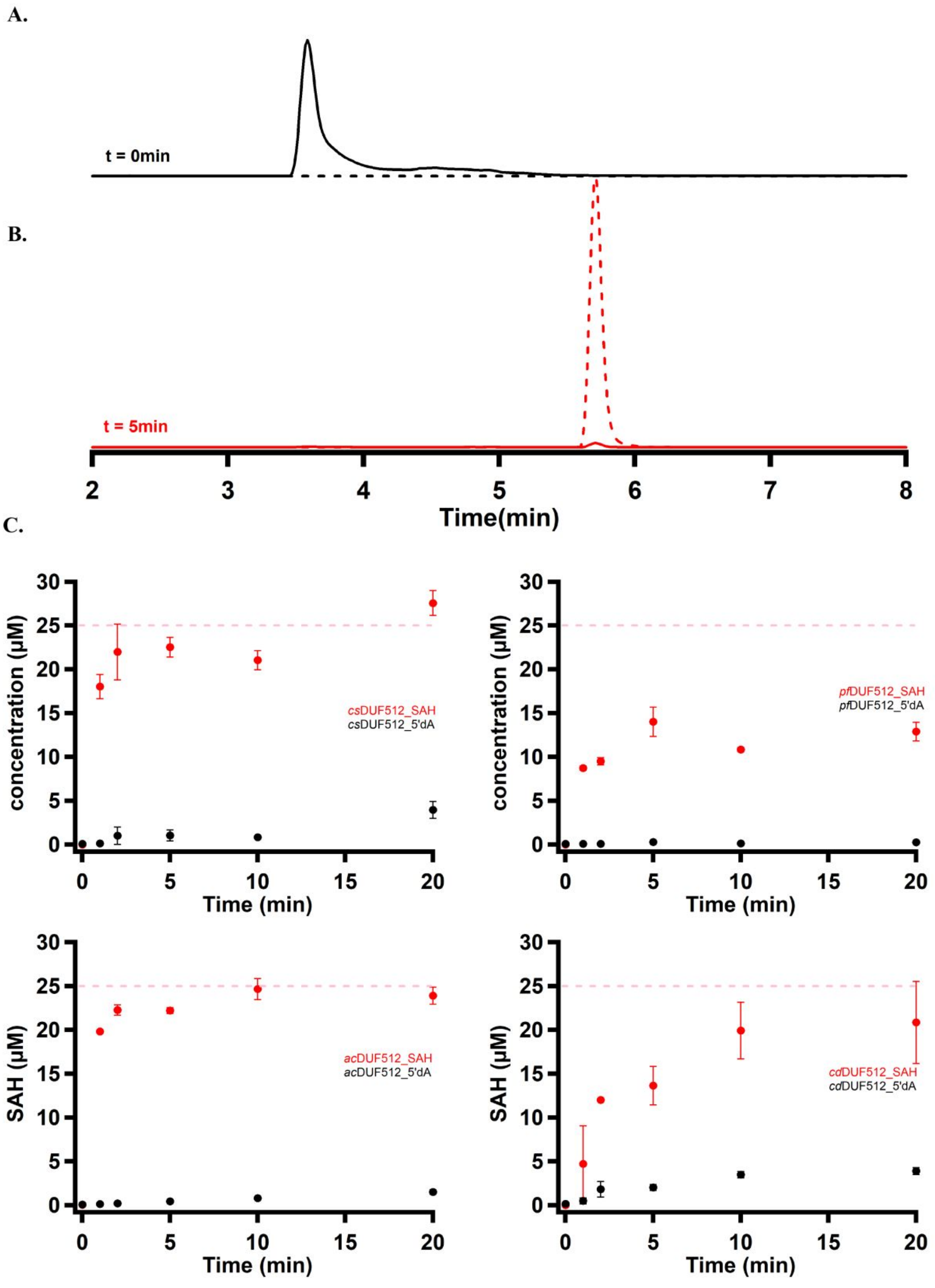

Figure S3. Cbl methylation reaction using *cs*DUF512, SAM, and titanium (III) citrate. **A.** reaction before adding titanium (III) citrate; **B.** *cs*DUF512 (50 µM) was incubated at room temperature with 1 mM SAM and 1 mM titanium (III) citrate in 50 mM HEPES, pH 7.5 (total reaction volume: 50 µL). After 5 min, the reaction was quenched by adding methanol (100 µL). The resulting reaction mixture was centrifuged, and

the supernatant was analyzed by LC-MS. In both figures, the solid line represents the multiple-reaction monitoring (MRM) trace of OHCbl (*m/z* 664.9 → 635.8); the dashed line represents the MRM trace of MeCbl (*m/z* 673.0 → 665.3). **C.** Quantifications of SAH and 5′dA. DUF512 proteins (25 μM) were incubated at room temperature with 1 mM SAM in 50 mM HEPES pH 7.5, 100 mM KCl, and 90 μM tryptophan as an internal standard. The reactions were initiated by adding titanium (III) citrate to 1 mM. 60 μL MeOH quenched reaction mixtures (30 μL) before initiation or at 1, 2, 5, 10, and 20min. Quenched reaction aliquots were centrifuged, and the supernatant was analyzed by LC-MS. In each figure, red dots represent SAH, and black dots represent 5′dA. The pink dashed line represents the concentration of DUF512 proteins used for the reaction, which was 25 μM.

A.

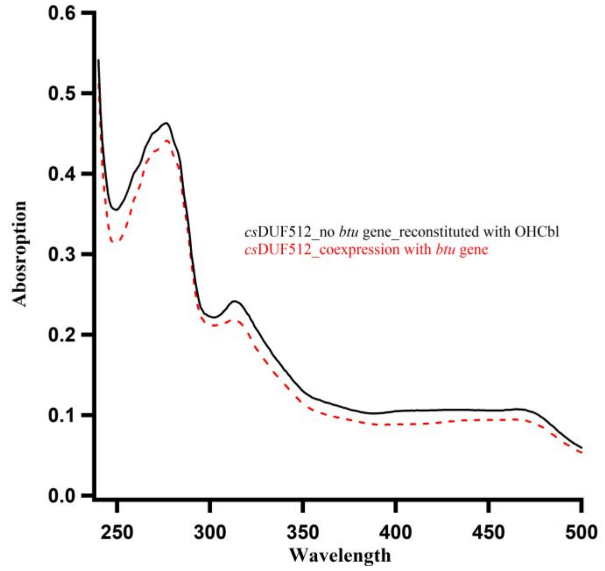

B.

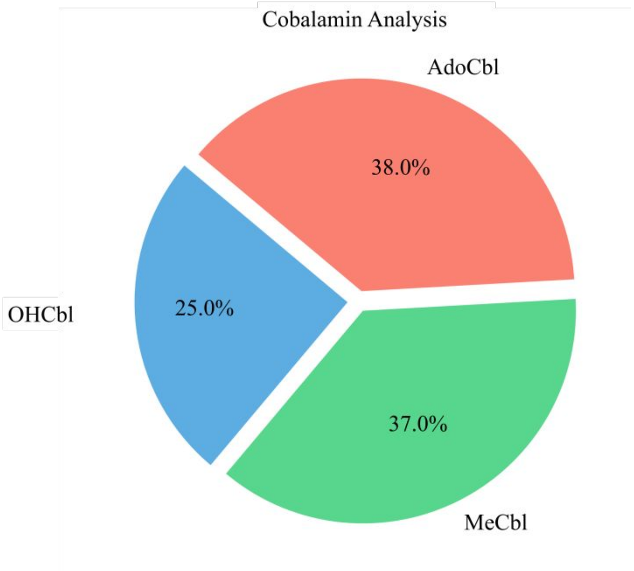

Figure S4. A. UV-vis spectrum of *cs*DUF512 coexpressed with *btu* genes (red dashed line) and without *btu* genes but reconstituted with OHCbl (black solid line); **B.** Cobalamin analysis shows a mixture of OHCbl, MeCbl, and AdoCbl is presented in *cs*DUF512 coexpressed with *btu* genes.

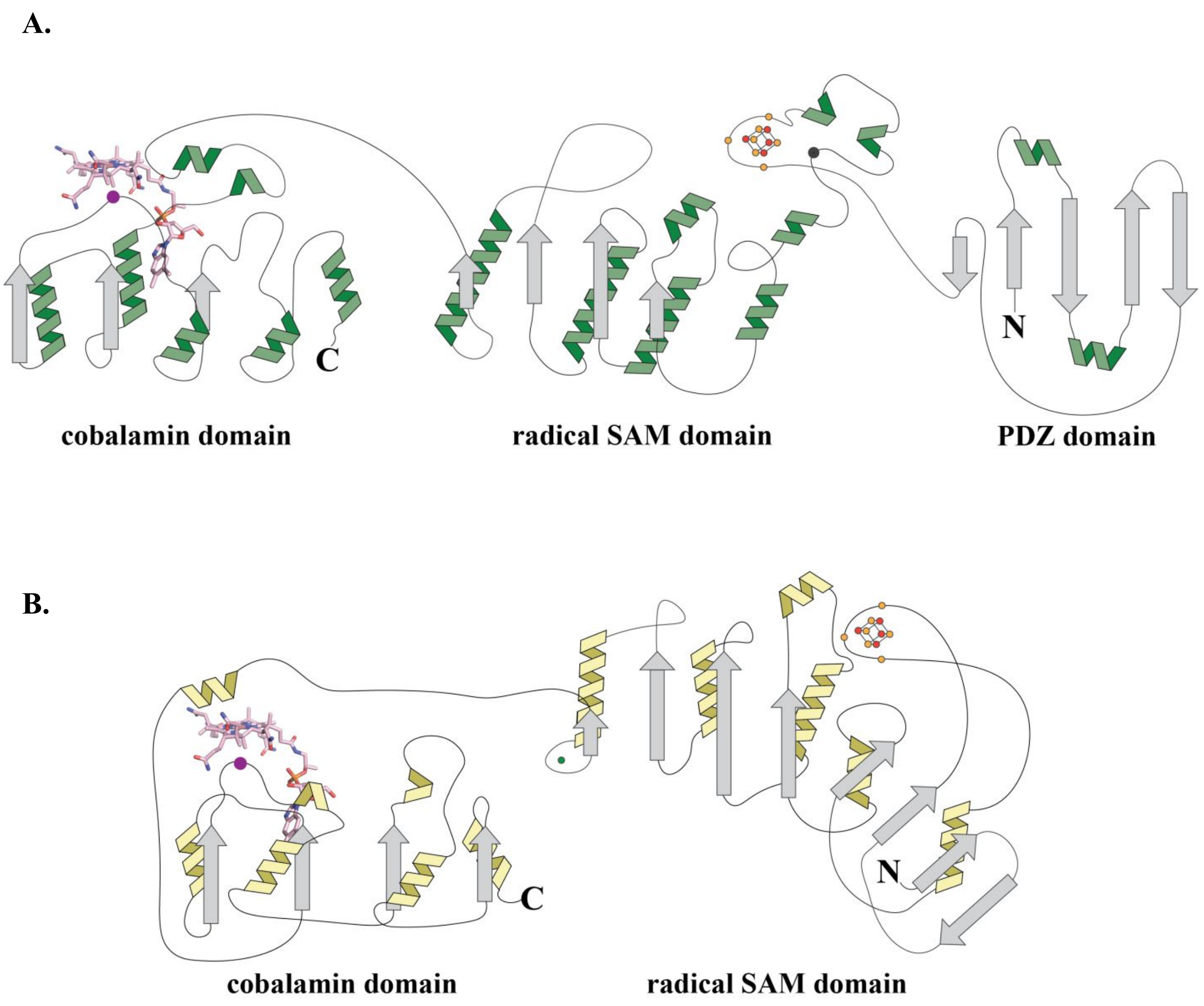

Figure S5. A. Topology diagram of *cs*DUF512 depicting domains. The Cbl is portrayed in sticks, and the lower axial Leu366 is shown as a purple dot. Three yellow dots surrounding the iron-sulfur cluster represent cysteine residues coordinating the cluster. The black dot represents Asp112, which coordinates the unique iron of the iron-sulfur cluster; **B.** Topology diagram of *pf*DUF512 depicting domains. The Cbl is portrayed in sticks, and the lower axial Leu286 is shown as a purple dot. Three yellow dots surrounding the iron-sulfur cluster represent cysteine residues coordinating the cluster. The green dot represents a potassium ion.

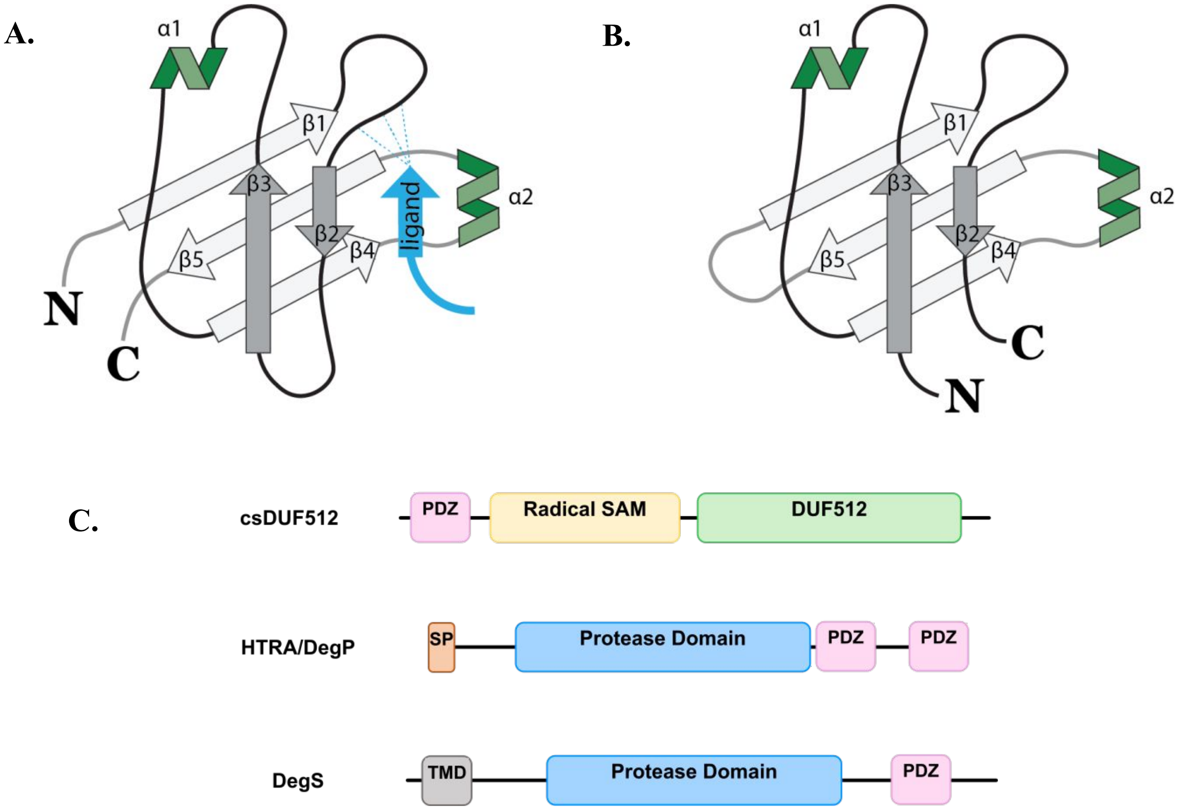

Figure S6. Topology diagram of PDZ domains of **A.** human CASK/LIN-2 (PDB ID:1KWA, organism: *Homo sapiens*);[1] **B.** *cs*DUF512. For comparison, all the secondary structures are numbered from N- to C-terminal according to the arrangement of human CASK/LIN-2 (PDB ID:1KWA). All PDZ domains have similar folds but with various secondary structure connectivity. The N- and C-termini of PDZ domains are always near each other. The blue strand in **Figure S6A** represents the PDZ domain binding partner that inserts into the cleft created by α_2 and β_2. The Blue dashed line represents the hydrogen bond between the carboxylate binding loop and the carboxylate of the binding partner. **C.** The domain architectures of several bacterial PDZ-containing proteins. HTRA family, including DegP/Q/S, has their PDZ domain on the C-terminal. While, *cs*DUF512 has its PDZ domain on the N-terminal. SP refers to an N-terminal signal peptide in HTRA/DegP protein. TMD refers to a transmembrane domain in DegS protein.

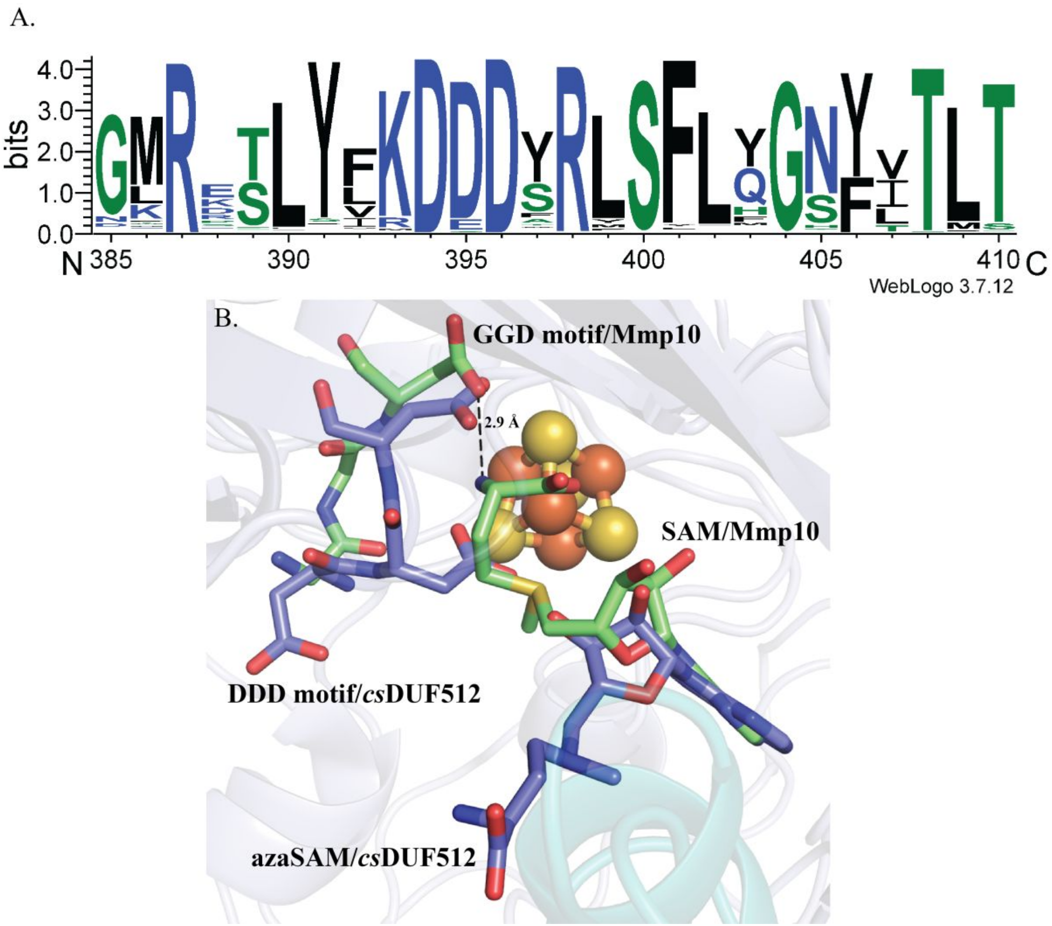

Figure S7. A. WebLogo sequence alignment of the PDZ-domain containing DUF512 proteins indicates that the DDD motif (Asp^{111}-Asp^{112}-Asp^{113} in the sequence of *cs*DUF512) is highly conserved. PDZ-domain containing DUF512 proteins (4097 proteins) were retrieved from all 6036 DUF512 proteins at InterPro (IPR007549). All PDZ-domain containing DUF512 proteins were subjected to multiple sequence alignment and visualized in WebLogo format;[2] B. DDD motif from *cs*DUF512 occupies similar position of the GGD motif from Mmp10 (PDB ID: 7QBS).[3] To generate the figure, *cs*DUF512 is aligned with Mmp10. For the clarity, both Cbl molecules and the cluster of *cs*DUF512 are not shown. The black dashed line indicate the hydrogen bond between the side chain of D90 to the amino group of SAM in the structure of Mmp10. GGD motif and SAM from Mmp10 are shown in green color. DDD motif and azaSAM from *cs*DUF512 are shown in blue color.

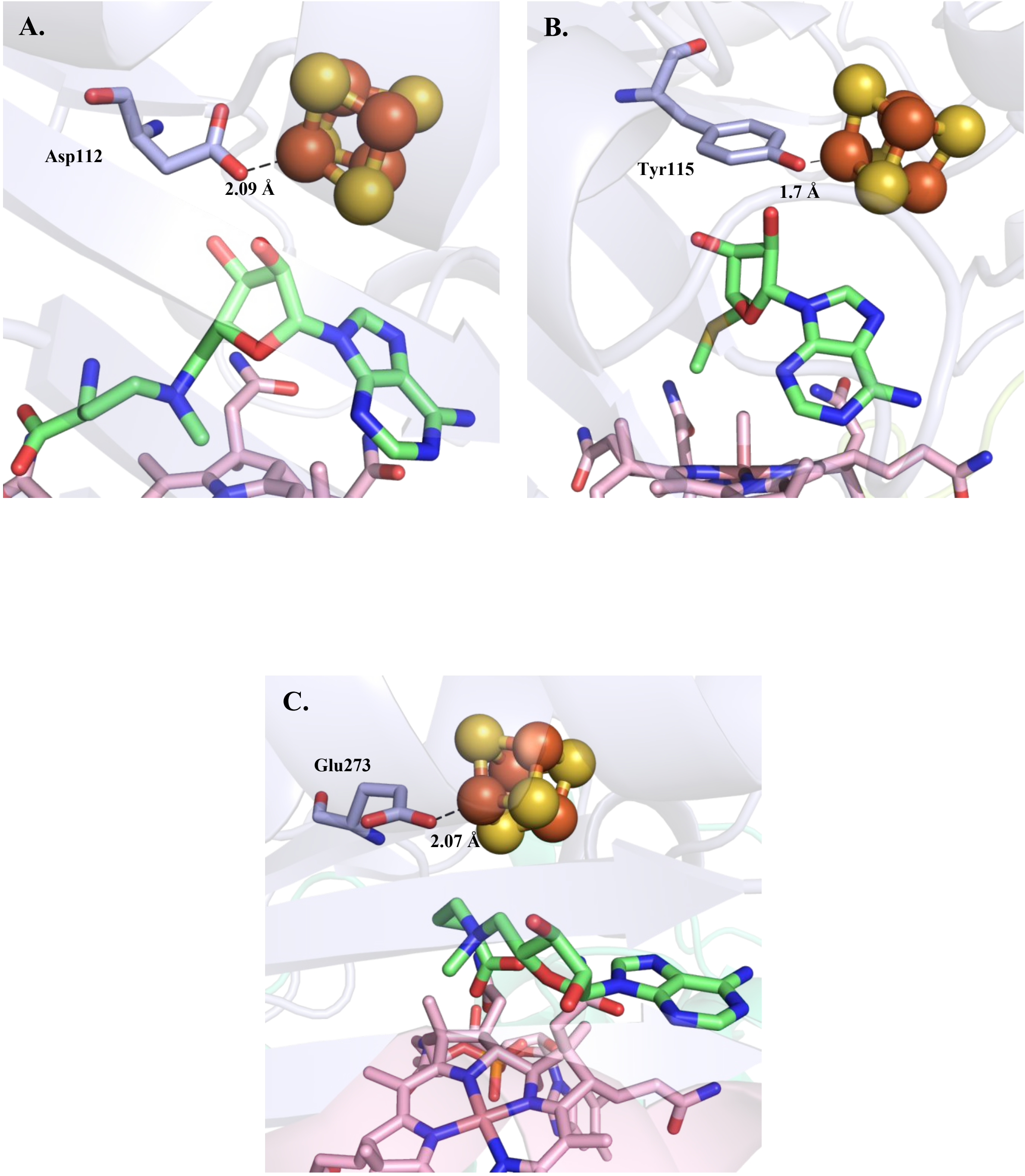

Figure S8. The distances between side chains of **A.** Asp112 to the unique iron (2.09 Å) in *cs*DUF512; **B.** Tyr115 to the unique iron (1.7 Å) in Mmp10 (PDB ID: 7QBU);[3] **C.** Glu273 to the unique iron (2.07 Å) in TsrM (PDB ID: 6WTF)[4] are shown with the black dashed line.

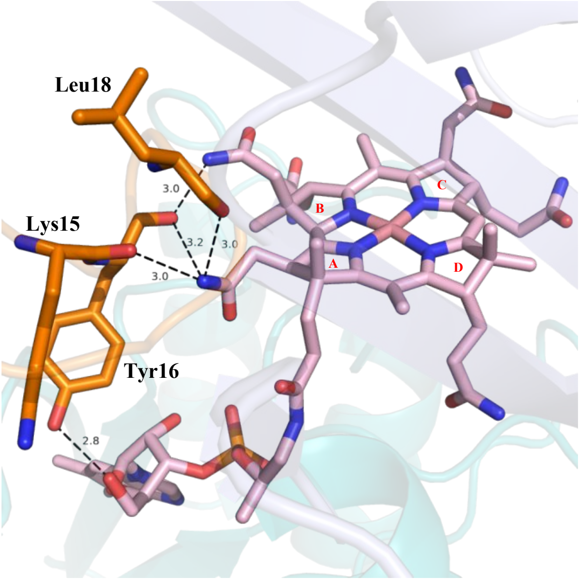

Figure S9. K15, Y16, and L18 (all shown in orange color) in L1 form hydrogen bonds with the acetamide side chains on rings A and B of cobalamin (pink) in *pf*DUF512 through their backbone carbonyl groups. The side chain of Y16 contacts the hydroxyl group of the ribose moiety of the DMB tail of cobalamin.

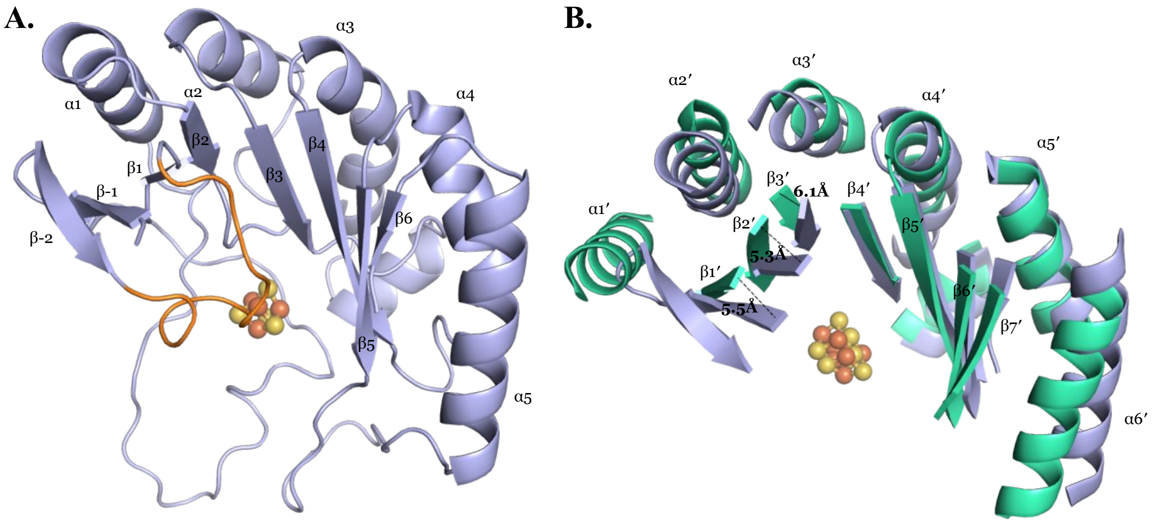

Figure S10. A. RS domain of *pf*DUF512. L1 is shown in orange; **B.** The superimposition of the RS domain of Mmp10 (green cyan) onto the RS domain (light blue) of *pf*DUF512. For clarity, loops in both structures were removed. The labeling of helices and strands is based on the $(\beta\alpha)_6$ TIM barrel of Mmp10. *pf*DUF512 has five βα repeating units in its TIM barrel structure, which align with the last five βα repeating units of Mmp10's TIM barrel. The N-terminal antiparallel β-sheet of *pf*DUF512 occupies the space of the first βα repeating unit of the $(\beta\alpha)_6$ TIM barrel.

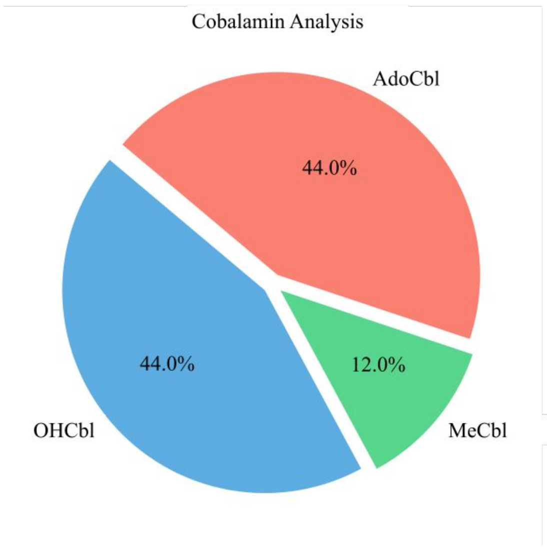

Figure S11. Cobalamin analysis shows a mixture of OHCbl, MeCbl, and AdoCbl is presented in *pf*DUF512.

A.

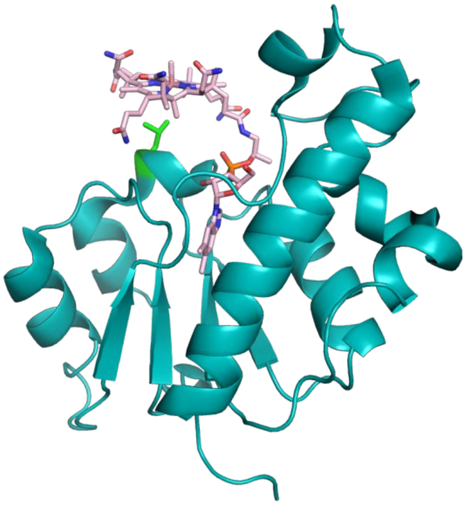

B.

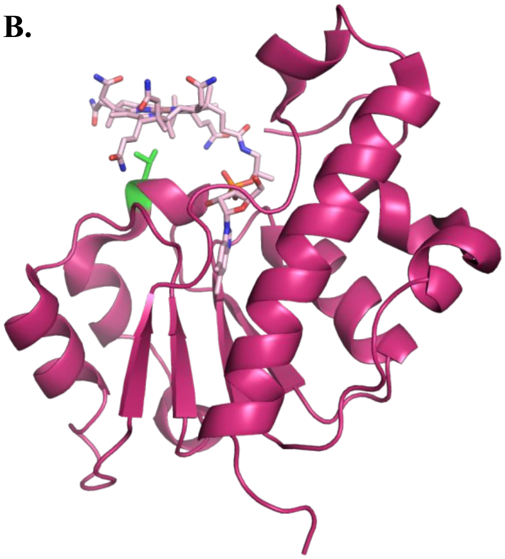

C.

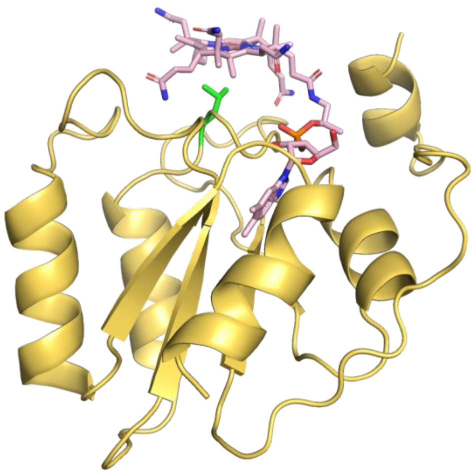

D.

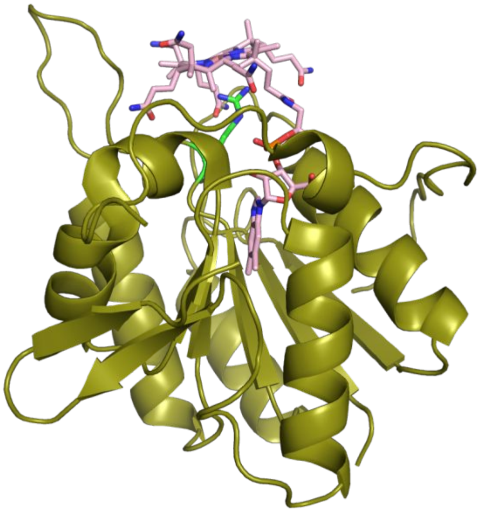

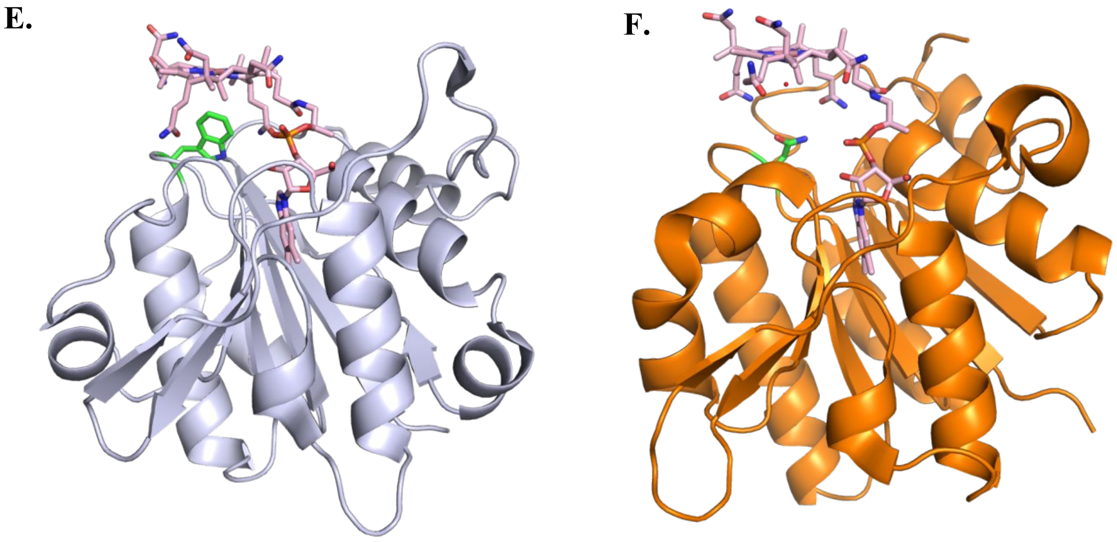

Figure S12. Cbl-domains of **A.** Mmp10 (PDB ID: 7QBS),[3] **B.** *cs*DUF512, **C.** *pf*DUF512, **D.** TsrM (PDB ID: 6WTF),[4] **E.** TokK (PDB ID: 7KDY),[5] and **F.** OxsB (PDB ID: 5UL4).[6] The cobalamin species are shown in pink. Amino acids in lower axial position of cobalamin (L322/Mmp10, L366/*cs*DUF512, L286/*pf*DUF512, R69/TsrM, W76/TokK, and water107-N186/OxsB) are shown in green.

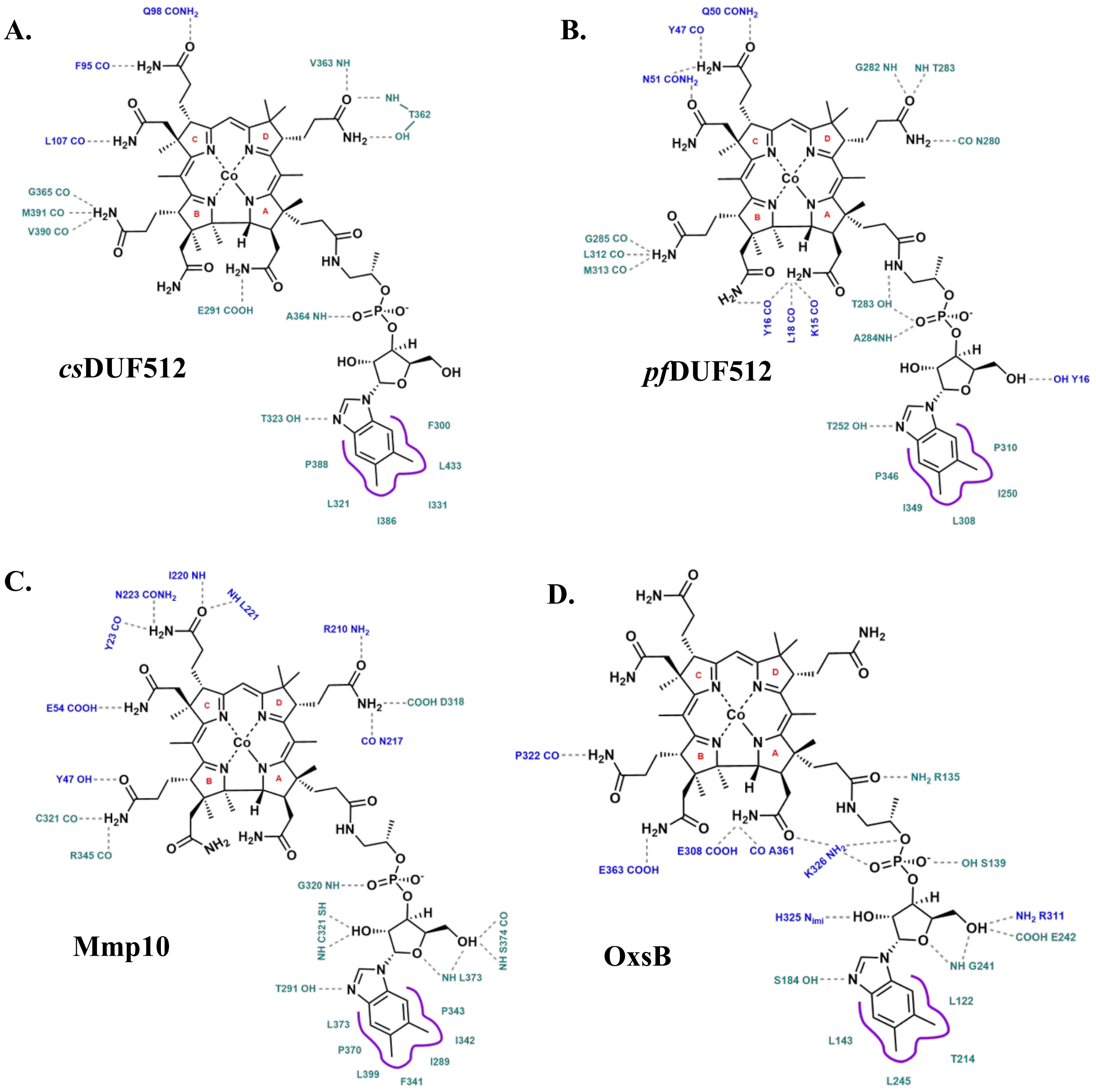
A.
csDUF512
B.
pfDUF512
C.
Mmp10
D.
OxsB

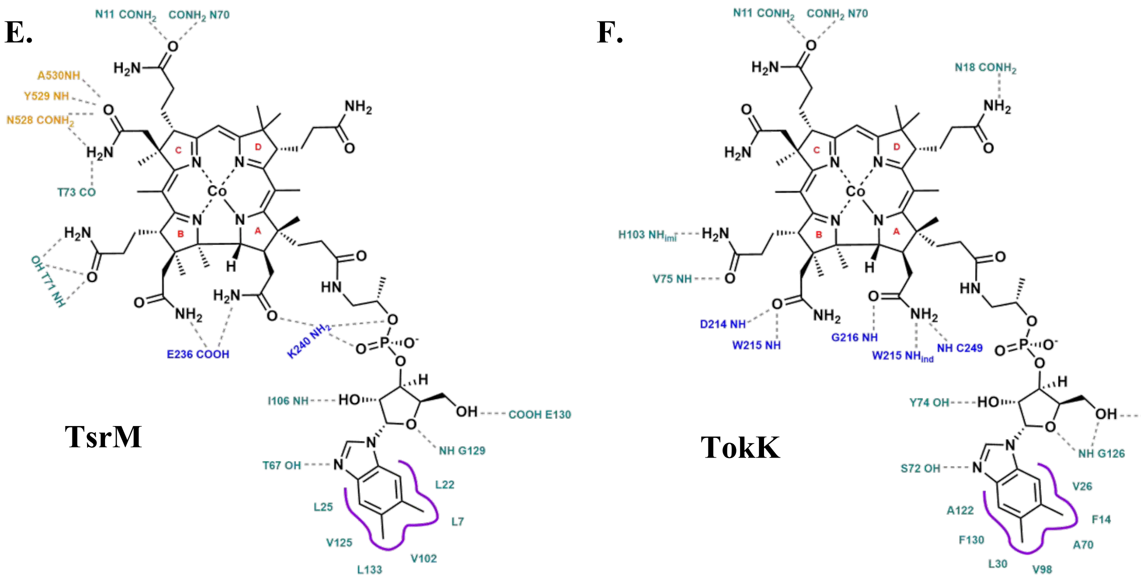

Figure S13. A. Direct H-bonding between cobalamin and *cs*DUF512, **B.** *pf*DUF512, **C.** Mmp10, (based on PDB ID: 7QBU),[3] **D.** OxsB (based on PDB ID: 5UL4),[6] **E.** TsrM (based on PDB ID: 6WTF),[4] and **F.** TokK (based on PDB ID: 7KDY).[5] Cobalamin is shown in black with its four pyrrole rings (A, B, C, and D) labeled in red. Residues in green belong to the Cbl-binding domains of each protein. The Cbl-binding domains of *cs*DUF512, *pf*DUF512, and Mmp10 are at the C-terminus, and the Cbl-binding domains of OxsB, TsrM, and TokK are at the N-terminus. Residues in blue belong to the RS domain of each protein. Residues in orange belong to the C-terminal domain of TsrM. The purple line shows the hydrophobic pocket for binding the DMB tail. Hydrogen bonds are depicted as gray dashed lines. The abbreviations for residues in the figure include: CO = carbonyl group of amide bond; NH = amino group of amide bond; OH = hydroxyl group in the side chains of serine, threonine, or tyrosine; COOH = carboxylate group in the side chains of glutamic acid or aspartic acid; $CONH_2$ = amide side chains of glutamine or asparagine; NH_2 = amino group of the side chains of arginine or lysine; SH = thiol group in the side chains of cysteine; NH_{imi} = nitrogen in the imidazole side chain of histidine; NH_{ind} = nitrogen in the indole side chain of tryptophan.

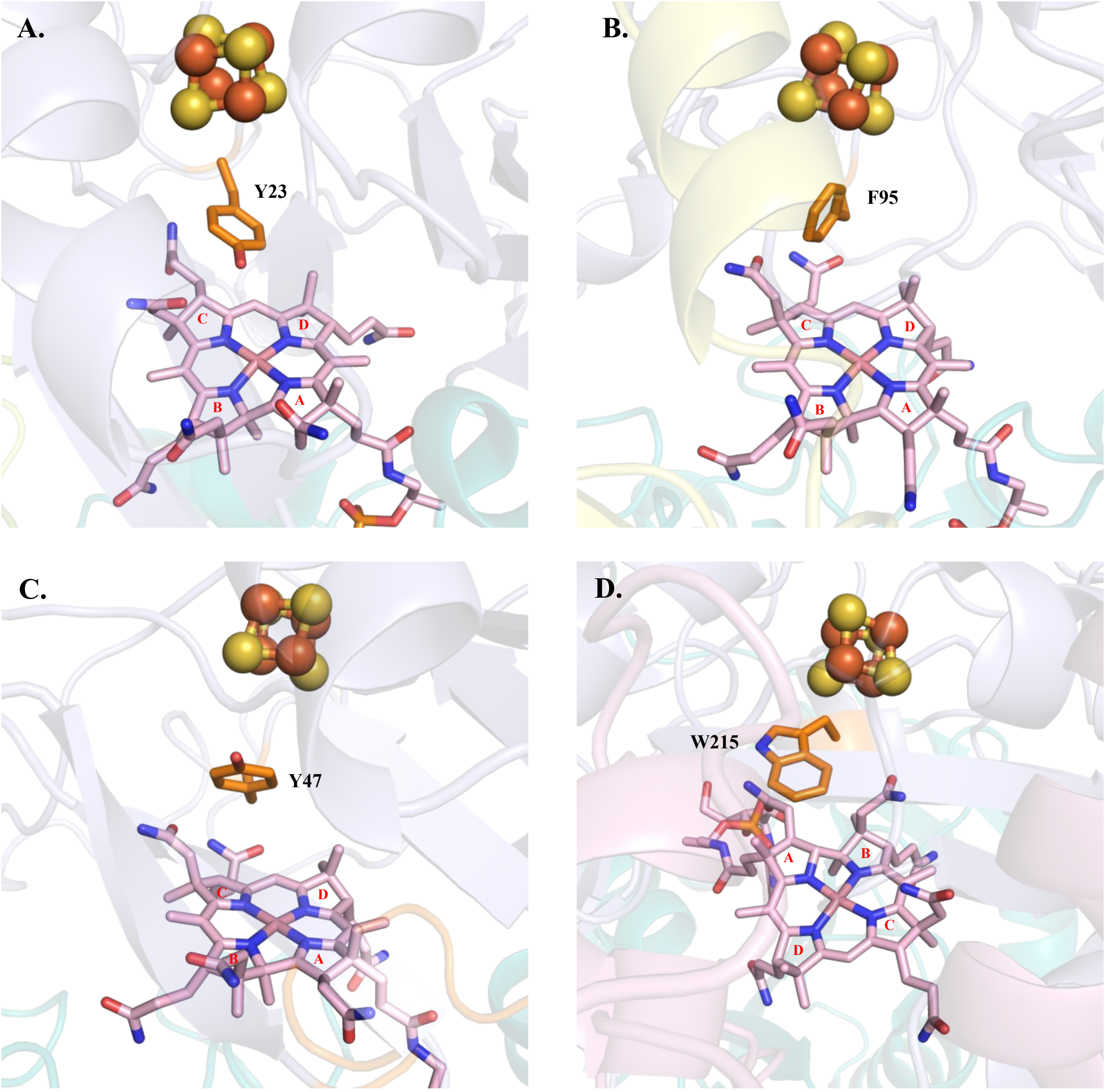
A.
Y23
C
D
B
A
B.
F95
C
D
B
A
C.
Y47
C
D
B
A
D.
W215
A
B
C
D

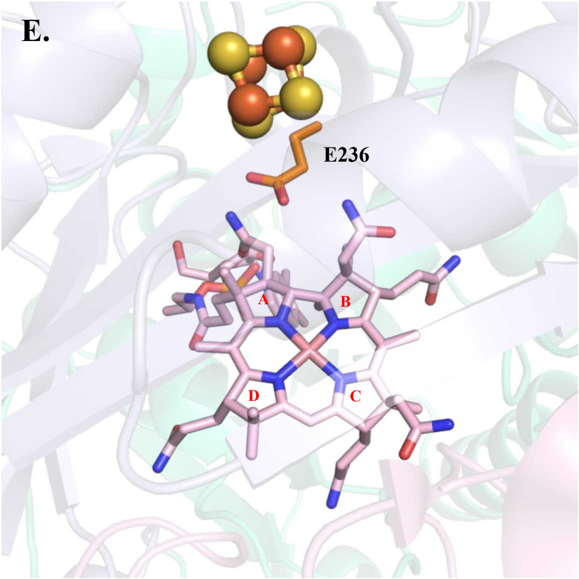

Figure S14. Active site structures showing the relative positions of cluster-Y23-cobalamin in **A.** Mmp10 (PDB ID: 7QBS),[3] **B.** cluster-F95-cobalamin in *cs*DUF512, **C.** cluster-Y47-cobalamin in *pf*DUF512, **D.** cluster-W215-cobalamin in TokK, (PDB ID: 7KDY)[5] and **E.** cluster-E236-cobalamin in TsrM, (PDB ID: 6WTF).[4] For clarity, SAM analogs or substrates are not shown: SAM for Mmp10, AzaSAM for *cs*DUF512, SAH for *pf*DUF512, AzaSAM/tryptophan for TsrM, and methionine/5′-dA/carbapenam substrate for TokK. The pyrrole rings of cobalamin are labeled as A, B, C, and D in red.

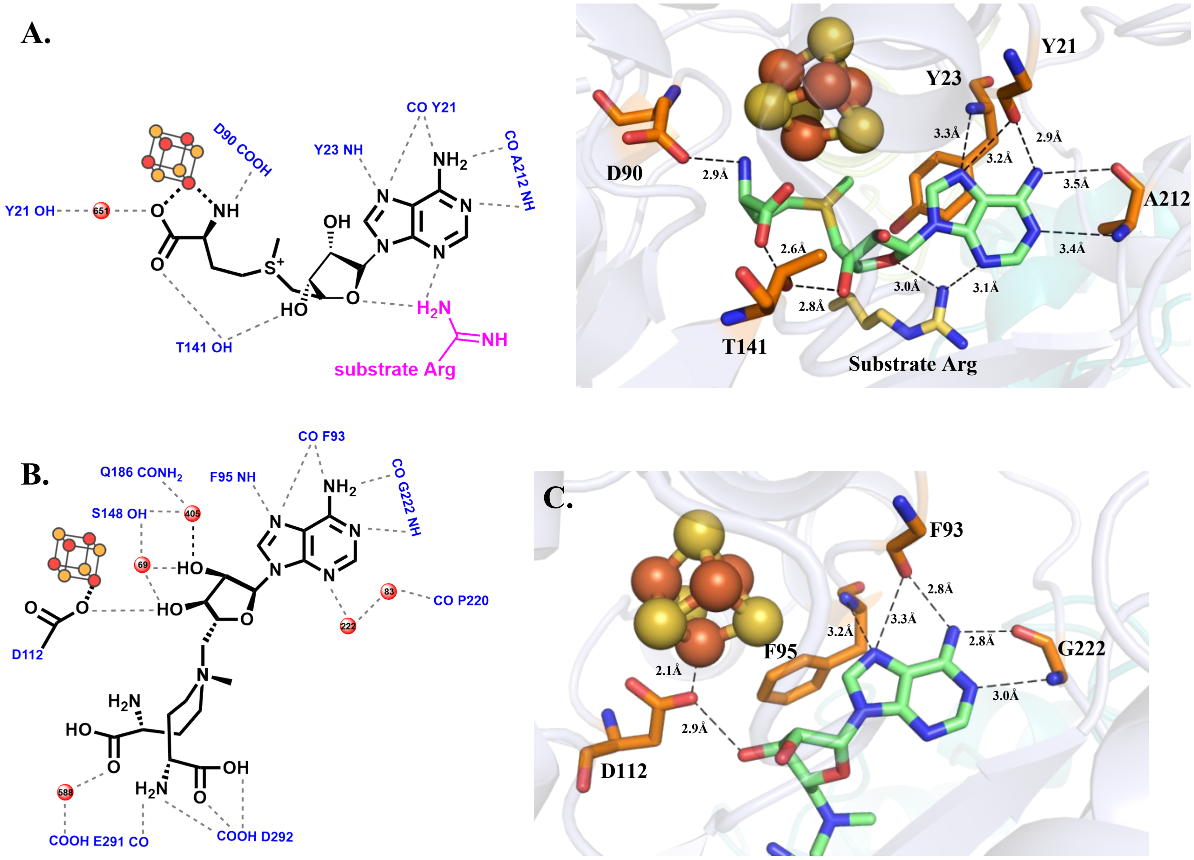

Figure S15. A. Interaction networks between SAM and Mmp10 (PDB ID: 7QBS) are presented as chemical (left) and pymol (right) structures. The side chain of Y21 is not shown for clarity; **B.** Interaction networks between AzaSAM and *cs*DUF512 are presented as chemical structures. **C.** Interactions between the adenosine moiety of AzaSAM and *cs*DUF512 presented as pymol structure. The side chain of F93 is not shown for clarity.

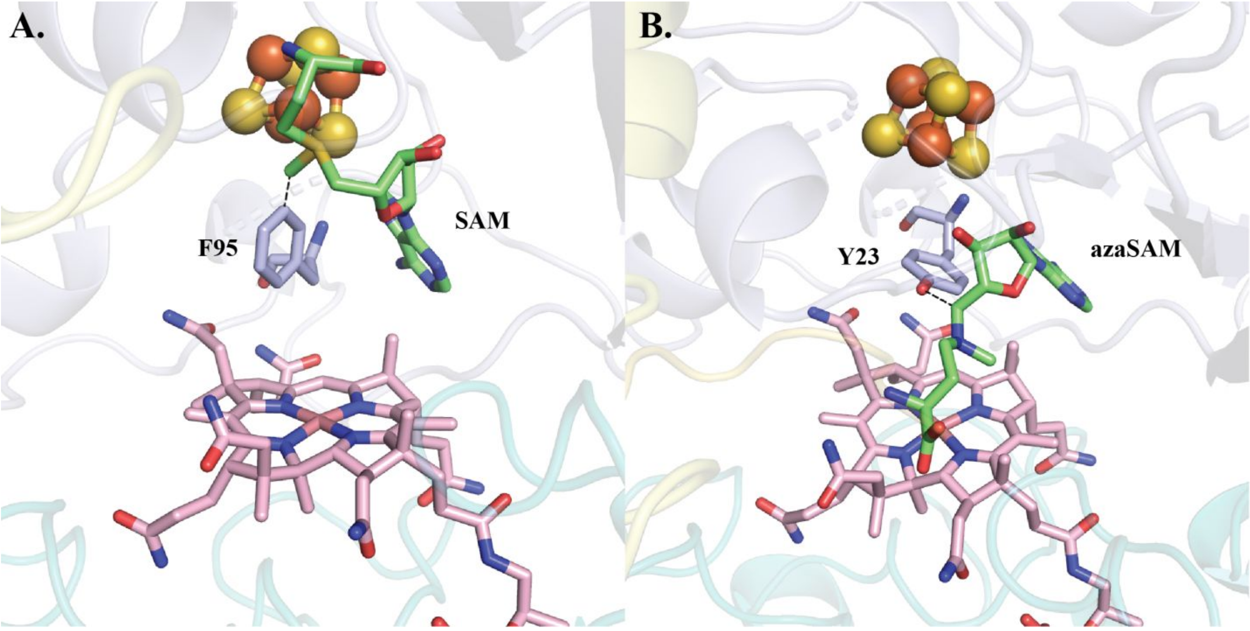

Figure S16. Superimposing the *cs*DUF512 structure with azaSAM to the Mmp10 structure (PDB ID: 7QBU)[3] with SAM shows that F95/Y23 is one of the residues distinguishing radical methylation from S_N2 methylation. The figures were generated by aligning two structures. For **A**, azaSAM from csDUF512 and Y23 from Mmp10 were deleted. Instead, SAM from Mmp10 and F95 from csDUF512 were shown; For **B**, SAM from Mmp10 and F95 from csDUF512 were deleted. Instead, azaSAM from csDUF512 and Y23 from Mmp10 were shown. In both figures, dashed lines indicate the clashes.

A.

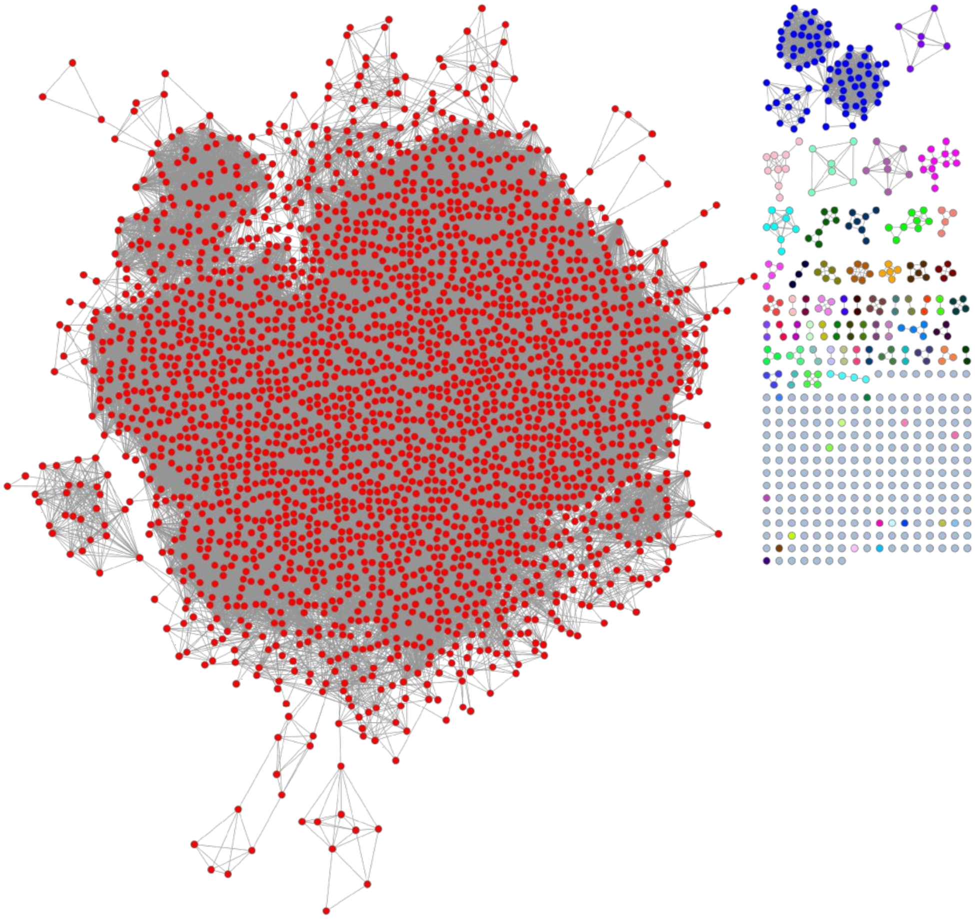

B.

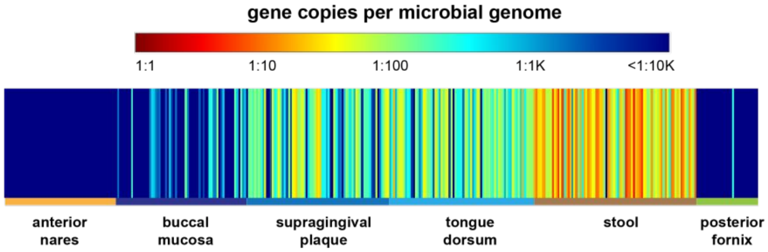

Figure S17. Enzyme Function Initiative (EFI) Analysis of DUF512 proteins in the human microbiome. A. SSN of DUF512 containing radical SAM proteins. The network is filtered to 50% sequence identity. B. Computationally-Guided Functional Profiling (CGFP) of the large DUF512 cluster (shown in red above). The DU512 genes are highly prevalent in the stool metagenomes and, thus, are connected to the human gut microbiome.

Sequence of the codon-optimized gene of the DUF512-containing protein from *Acinetobacter sp. CAG: 196_36_41* (UniProt ID: A0A1Q6TSJ3, *ac*DUF512) as supplied by GeneArt:

5′-

CATATGCCTGCAGTTGTTAGCAGCGTTGTTCAGGGTAGCATTGCAGATGAACTGGCAATTGA
AGAAGGTGATATTCTGCTGAGCATTGATGGTGAAAAACTGCTGGATATGATCGATTATCGCT
TCCTGTGCAAAAACGAATTTATCACCATCGAGATCCAGAAAAAAAACGGCGAAATTGAAGA
GATCGAGCTGGAAAAAGATTTCGATGAAGATCTGGGCATTGTGTTTGAAAGCGCAGTTTTTG
ATCGTGTTAAACCGTGTCTGAATAACTGCATCTTTTGTTTTGTTGCACAGCAGCCGAAAGGTC
TGCGTAAAACCCTGTATATCAAAGATGATGATTACCGCCTGAGCTATCTGCAGGGCACCTAT
ATTACCACCACCAATCTGAGCGAAAGCGATAAAGAACGTATTAGCCGTCTGCATCTGGGTCC
GTTTTATGTTAGCGTTCATACCACAAATCCGGATCTGCGTGTTAAAATGCTGCGTAATCTGAA
TGCAGGCAAAATCATGGATAATCTGCGCTGGTTTAAAGAAAACGAAATCCCGTTTCATACCC
AGATTGTTCTGTGTCCGGGTTATAATGATGGCAAAGAACTGCGTCGTACCCTGGAAGATCTG
AGCAGCCTGGGTGAAGCAGTTCTGAGTATTGCAATTGTTCCGGTTGGTGTTACCCAGTTTCGT
CAGAGCGAACTGAAAACCGTTAATGCCGTTATTGCACGTGAAACCATTGAAATTGCAAGCA
ATTATCCGAAAGCCTGCTGCTCCGATGAATTTTTTCTGCTGGCAGGTCAGGATATTCCGAGTG
CAGATTATTATGGTAATTTCAGCCAGCTGGATGATGGTGTTGGTAGCCTGCGTACCCTGATG
GATGATTTTGATACCTTTGAACTGCCGGATCGTCTGGGTAAAAAACTGTCAATTGCATTTGC
ATGTAGCGTTGCAGCAGAAAGCGCCTTTAAATACATTAGCAGCAAACTGAACAAAATCAAA
AACCTGCATACCAGCGTTCATCCGGTTAAAAGCACCTATTGGGGTAAAAACATTACCGTTGC
AGGTCTGATTACCAGCGAGGATCTGATTAATGCCATTATCAATATCAACGCCGACTATATTA
TCGTGCCGAGCATTATGCTGAAACCGTATAGCGACCTGTTTCTGGATGGTAATAGCCTGGAT
TATGTGATTGAAAAAAACCGGCAAAAACATCTTTGTGGCCGAAAACAATTATAGCCTGTGCGA
AGTTATTGACCTGATCAACGAATATGTGTAACTCGAG-3′

Sequence of the codon-optimized gene of the DUF512-containing protein from *Clotridioides difficile ATCC 9689 = DSM 1296* (UniProt ID: A0A125V8J6, *cd*DUF512) as supplied by GeneArt:

5′-

CATATGGAAGTGAACGTGAAGAAGGTGAACAACATTATCAGCAAAGTGTATAAGGATTCGA
TCGCCGAAGAAATGGGTATTGAAGTTGGTGATCTGCTGATTAGCGTTAATGAACAGCCGATC
CATGACATTATCGAATATCGTTTTCTGCTGAGCGACGAATATCTGGATGTTGAGATTCAGAA
AAAAGATGGCGAGGTGTATATCTACGAGATCGAGAAAGATTATGATGAGGATCTGGGTGTC
GAATTTACCAATCCGATTATTGATCAGGCAAAAAGCTGCCGCAATAAATGCATGTTTTGCTT
TATCGATCAGCTGCCGGAAGGTATGCGTGAAACCCTGTATTTCAAAGATGATGATAGCCGTC
TGAGCTTTCTGCAGGGTAATTTTGTTACCCTGACCAATATGAGCGAAGAGGATATTAACAAC
ATCATCAAATATCGCATCAGCCCGATTAACATTAGCGTTCATACCACCAATCCGGAACTGCG
TCAGAAAATGATTAGCAACAAATTTGCCGGTAAACTGTACGGCATTATGAAACGTCTGGCAG
ATGCACATATTGAAATGAATTGTCAGATTGTTCTGTGCCCTGGTATTAACGATGGTAAAGAA
CTGGATCGCACCATTAAAGAGCTGGCACAGCTGTATCCGTATGTTAATANNNNTGCAATTGT
TCCGGTTGGCATTACCAAACATCGTGAAAATCTGGTGGAACTGAACATCTTCAATGATAAAA
GCGCCAGCAAAACCATTGAGCAGATTCATCAGATCCAGCAGAAATACCTGGAAAAACTGGG
CACCCGTTTTGCATTTCTGAGTGATGAATTCTATATCCTGAGCAATAGCGAACTGCCTGGCTA
TGAAGAATATGAAGGTTTTCTGCAGTTTGAAGATGGTGTGGGTATGATTCGTAAACTGAAAA
CCGAAATCGAAGAGTACCTGAATATTCTGCCGGAAAATATTCTGAAACGCGAAAAGAAAGT
TTCCATTGCAACCGGTCATAGCGCCTATGAATTTATTCAGAGCATGGCCGATGCAATGATGG
ACAAATTCAAAAATCTGCAGGTCAACGTGTACGAGATCAAAAACAAATTTTTCGGCGAAAC
CATTACCGTTAGCGGTCTGCTGACCGCAAAAGATCTGAAAGAACAGCTGGAAGATAAAGAA
TTAGGTGAGGCACTGTATATTACCCGTAGCATGCTGAAAGCAGATGAAGAAATTTTCCTGGA
TAACATCGAACTGAACCAACTGGAAGAACTGATGCGCATTAAAATCATTCCGTGTCTGAATG
AGGGCAAAGATTTCGTGGATAAAATCCTGAAATAACTCGAG-3′

Sequence of the codon-optimized gene of the DUF512-containing protein from *Clostridium sporogenes* (UniProt ID: A0AAE4Z2Q4, *cs*DUF512) as supplied by GeneArt:

5′-
CATATGAAGAAAGAAATCCTGAAAGTGGAACGTGGTAGCATTGCAGAAGAACTGGAA
ATTGAAAAAGGCGATTTTCTGCTGAGCATCAACAACAAAGAAGTGAAAGATATCATCGAT
TACAAATTTCTGGTGTGCGACGAATATCTGGAAGTGGAAATCGAGAAAAGCAATGGTGAA
CTGTGGGAACTTGAGATCGAGAAAGATTATGATGAAGATCTGGGCATCGAATTCAAAGCA
GCAATTCTGGATGTTCCGCAGCGTTGTCATAATAACTGTCTGTTTTGCTTTATTGACCAG
CTGCCGAAAGGTATGCGTAAAACCCTGTATTTCAAAGATGATGATAGCCGTCTGAGCTTT
CTGCAGGGTAATTTTCTGACCCTGACCAACATGAAAGATGAAGATATTGAACGCATCATC
AACTACAAAATCAGCCCGATTAACATTAGCGTGCATACCACCAATCCGGAACTGCGTGTT
GAACTGCTGAATAATCGTTTTGCCGGTAACATTTATGAGCGCATGAAAAAGCTGGCCGAA
GGTGGCATTAAAATGAATTGTCAGGTTGTTCTGTGTCCGGGTCTGAATAATGCCGAAGAA
CTGAAACGTACCATCGAAGATCTGTATGCACTGTATCCGCAGGTTGAAAATCTGGCAGTT
GTGCCGATTGGTGTTACCAAATTTCGTGAAGGTCTGTATCGTTTTGAACTGTTTAACAAA
GAAACCGCGAACAAAGAACTTGACATGGTGGAAGAATACCAGAACAAGTTCATTAAAGAA
ATCGGCAAACCGTTTGTGCGTCTGTCCGATGAATTCTATGTTATTGCAGAACGCGAAATC
CCGAAAGAAGAATTTTACGACGGTTTTCATCAGCTGGAAGATGGTGTTGGTGTGATTCGT
ATTTTTCGCAACAACATCAAGAACAACGTGAAGAAACTGAGCACCAAAGTGAAAGGTAGC
TTTAGCCTGATTACCGGTCAGAGCGCATATAAAGAAATTCTGGAAGCAAGCCGCATTATC
AACAACTATAACAACGATATCAACATCGAGGTGATCAAAATCGACAACAACTTTTTCGGT
AAAACCATTACCGTTGCAGGTCTGATTACAGCCAACGATATTATTGAACAGACCCAAGAG
AAAAACCTGGGCAAATATGTTATTATCCCGGATGTTATGCTGCGCAAAGGTTATGAACTG
GCAGATATTAGCGAACAGGTGTTTCTGGATGATGTTACCCTGAAAGAACTGAGCAAAAGC
CTGAAACGTGAAATCCTGGTTTGTGATTATACCGGTGAGGATCTGATTGATATCATCAAC
AAACATAGCCGTGAAGAATAACTCGAG-3′

Sequence of the codon-optimized gene of the DUF512-containing protein from *Pyrococcus furiosus (strain ATCC 43587)* (UniProt ID: Q8U2I5, *pf*DUF512)

5′-

CATATGTATGAACTGACCGAGGATTTCAAACTGCGCAAAATCACCAAATATGAACTGGATG

GTGTTGATGAACGTGAAGATCTGCTGGTTATTCCGCCTAGCAGCAAAGCAGGTCCGTGTGGT

AATGGTTGTCTGTTTTGTTATCTGCTGCAGAATCCGCCTGAAATGATTTATCGTGTTGCACGT

CATGATACCCTGAATGATCCGACACTGGAAGAACGTATTCGTTATGCACGTAAACATTATGA

TCTGTGGATTCGTGTTACCGATACCAGCGGTAATGTGAAATTTGATGAAAACCGCATCAAAA

GCCTGTATGAAGCAGGTCTGGATGAAATTCAGATTAGCGTTCATACCACCAAAAAAGACGT

GCGCATTAAACTGATGCGTAATCGTCATGCCGGTAAACTGATTGATCTGCTGCCGCTGGTTG

CAAAACATTTTCGTACCATTGCAGATATTATTCTGACCCCTGGTTTTAACGTGGATGATATTG

GCGAAATTATCGAGGATCTGGATAGCATGGGTGTTCATGAAGTTCGTCTGTTTCCGGTTGGT

GTTACCAAATATAACCGCTTTGAAATTCGTCCGCTGACCAAAGAAGAACTGAGCTACGTTAA

AGAAGTTGCCCTGGAAAAAGATAAAGAGCTGGGTATCAAAGTTGTGATCCCTCCGATTTTTC

TGGCACTGCTGGGTGAATTTACCACCGGTCTGGAACCGTTTAACATTGAACCGGAATTTCCG

ACCTATATCTTTACCGGTGAACTGGCATATCCGGAAATGAAACGCCTGTTTCCGCGTATTAA

AGTGGTGATGGTGAAAAACGAATTTTTTGGCGGTAATATTGGCACCGCAGGTCTGCTGACCG

GTCGTGATGTTCTGCGTGAAGTTGAACGTCTGCCGGAAGTTGATTTTGGTCTGATTCTGCTGC

CTGAACTGATGTTTTATGGTGATATGACCCTGGATGGTTGGCGTCGTCAGGACCTGTTTAGC

AAAATTCTGATTGAGAAAGGCTACATCGTTGAAACCGCACTGGAACCGACCGAAATTCCGA

AAGTTATTGAAAAAATCAGCCTGTAACTCGAG-3′

Table S1. Crystallographic data table for *cs*/*pf*DUF512 structures obtained.

	*cs*DUF512	*pf*DUF512
**Data collection**		
Wavelength	1.03317	1.03768
Resolution range	50 - 1.68 (1.71 - 1.68)	50 - 1.96 (1.98 - 1.95)
Space group	$P\ 1\ 2_1\ 1$	$P\ 2\ 2_1\ 2_1$
Cell dimentions		
a, b, c (Å)	56.67, 59.30, 81.06	50.88, 80.45, 98.43
α, β, γ (°)	90, 109.8, 90	90, 90, 90
Total reflections	85596 (5440)	33217 (2176)
Unique reflections	57383 (5434)	28296 (2175)
R_{sym} or R_{merge} *	0.058 (0.524)	0.070 (0.610)
R_{pim}	0.025 (0.226)	0.040 (0.339)
I / σ *	27.2 (3.4)	16.0 (2.3)
$CC_{1/2}$ *	0.994 (0.905)	0.982 (0.725)
Completeness (%) *	99.9 (99.2)	98.7 (99.6)
Redundancy *	6.4	3.7
**Refinement**		
Resolution (Å)	1.68	1.96
Reflections used in refinement	82561	28289
R_{work} / R_{free}	0.15 / 0.20	0.16 / 0.21
Number of non-hydrogen atoms	4475	3383
Macromolecules	3699	2946
OH-cobalamin	91	91
Fe/S cluster	8	8
Imidazole	10	N/A
azaSAM	54	N/A
SAH	N/A	26
5′-dA	N/A	18
glycerol	6	N/A
Boric acid	12	N/A
potassium	N/A	1
water	497	303
B-factors (Å^2)		
Macromolecules	19.10	23.34
OH-cobalamin	10.9	18.4
Fe/S cluster	9.8	25.9
Imidazole	31.1	N/A
azaSAM	21.2	N/A
SAH	N/A	30.0
5′-dA	N/A	55.7
glycerol	35.8	N/A
Boric acid	30.4	N/A
potassium	N/A	15.5
water	29.5	29.8
RMS deviations		

Bond lengths (Å)	0.012	0.011
Bond angles (º)	1.42	1.09
Clashs score	3.58	4.06
Rotamer outliers (%)	1.20	0.62
Ramachandran		
Most favored (%)	98.4	97.2
Allowed (%)	1.6	2.8
Outliers (%)	0	0
Number of TLS groups	1	2
PDB accession code	9CG1	9CG2

*represents the (highest resolution shell) **all datasets result from a single protein crystal

1. Daniels, D. L.; Cohen, A. R.; Anderson, J. M.; Brunger, A. T., Crystal structure of the hCASK PDZ domain reveals the structural basis of class II PDZ domain target recognition. *Nat Struct Biol* **1998,** *5* (4), 317-25.
2. Crooks, G. E.; Hon, G.; Chandonia, J. M.; Brenner, S. E., WebLogo: a sequence logo generator. *Genome Res* **2004,** *14* (6), 1188-90.
3. Fyfe, C. D.; Bernardo-Garcia, N.; Fradale, L.; Grimaldi, S.; Guillot, A.; Brewee, C.; Chavas, L. M. G.; Legrand, P.; Benjdia, A.; Berteau, O., Crystallographic snapshots of a B(12)-dependent radical SAM methyltransferase. *Nature* **2022,** *602* (7896), 336-342.
4. Knox, H. L.; Chen, P. Y.; Blaszczyk, A. J.; Mukherjee, A.; Grove, T. L.; Schwalm, E. L.; Wang, B.; Drennan, C. L.; Booker, S. J., Structural basis for non-radical catalysis by TsrM, a radical SAM methylase. *Nat Chem Biol* **2021,** *17* (4), 485-491.
5. Knox, H. L.; Sinner, E. K.; Townsend, C. A.; Boal, A. K.; Booker, S. J., Structure of a B(12)-dependent radical SAM enzyme in carbapenem biosynthesis. *Nature* **2022,** *602* (7896), 343-348.
6. Bridwell-Rabb, J.; Zhong, A.; Sun, H. G.; Drennan, C. L.; Liu, H. W., A B(12)-dependent radical SAM enzyme involved in oxetanocin A biosynthesis. *Nature* **2017,** *544* (7650), 322-326.
